# An intra-specific consensus genetic map of pigeonpea [*Cajanus cajan* (L.) Millspaugh] derived from six mapping populations

**DOI:** 10.1007/s00122-012-1916-5

**Published:** 2012-07-08

**Authors:** Abhishek Bohra, Rachit K. Saxena, B. N. Gnanesh, Kulbhushan Saxena, M. Byregowda, Abhishek Rathore, P. B. KaviKishor, Douglas R. Cook, Rajeev K. Varshney

**Affiliations:** 1 International Crops Research Institute for the Semi-Arid Tropics (ICRISAT), Patancheru, 502324 India; 2 Osmania University, Hyderabad, 500007 India; 3 University of Agricultural Sciences, Bengaluru (UAS-B), 560065 India; 4 University of California, Davis, CA 95616 USA; 5 CGIAR Generation Challenge Programme (GCP), c/o CIMMYT, 06600 Mexico DF, Mexico

## Abstract

Pigeonpea (*Cajanus cajan* L.) is an important food legume crop of rainfed agriculture. Owing to exposure of the crop to a number of biotic and abiotic stresses, the crop productivity has remained stagnant for almost last five decades at ca. 750 kg/ha. The availability of a cytoplasmic male sterility (CMS) system has facilitated the development and release of hybrids which are expected to enhance the productivity of pigeonpea. Recent advances in genomics and molecular breeding such as marker-assisted selection (MAS) offer the possibility to accelerate hybrid breeding. Molecular markers and genetic maps are pre-requisites for deploying MAS in breeding. However, in the case of pigeonpea, only one inter- and two intra-specific genetic maps are available so far. Here, four new intra-specific genetic maps comprising 59–140 simple sequence repeat (SSR) loci with map lengths ranging from 586.9 to 881.6 cM have been constructed. Using these four genetic maps together with two recently published intra-specific genetic maps, a consensus map was constructed, comprising of 339 SSR loci spanning a distance of 1,059 cM. Furthermore, quantitative trait loci (QTL) analysis for fertility restoration (Rf) conducted in three mapping populations identified four major QTLs explaining phenotypic variances up to 24 %. To the best of our knowledge, this is the *first* report on construction of a consensus genetic map in pigeonpea and on the identification of QTLs for fertility restoration. The developed consensus genetic map should serve as a reference for developing new genetic maps as well as correlating with the physical map in pigeonpea to be developed in near future. The availability of more informative markers in the bins harbouring QTLs for sterility mosaic disease (SMD) and Rf will facilitate the selection of the most suitable markers for genetic analysis and molecular breeding applications in pigeonpea.

**Electronic supplementary material:**

The online version of this article (doi:10.1007/s00122-012-1916-5) contains supplementary material, which is available to authorized users.

## Introduction

Pigeonpea [*Cajanus cajan* (L.) Millspaugh] is the fifth most important pulse crop in the world and represents an important component of semi-arid and sub-tropical farming systems (Shanower et al. 1999). Pigeonpea is a diploid species (2n = 2*x* = 22) and its genome comprises of 833.1 Mbp arranged into 11 pairs of chromosomes (see Varshney et al. 2012). Globally, it is cultivated in 4.6 Mha with a production of 3.49 Mt. Nearly 70 % of the pigeonpea production and 74 % of the pigeonpea area is in India. Pigeonpea is a hardy and drought tolerant crop assuring sustainable returns from marginal lands with minimal inputs, hence it is considered as a very suitable crop for subsistence agriculture. Pigeonpea seeds contain about 20–24 % protein and reasonable amounts of essential amino acids making it an important source of dietary protein, mainly in vegetarian-based diets.

Pigeonpea production has shown an increasing trend in worldwide harvested area from 2.7 Mha (1961) to 4.6 Mha (2009) (FAO 2009, http://faostat.fao.org/). However, no increase has been observed in its productivity, which in the past five decades remained stagnated at around 750 kg/ha. To overcome the existing yield barriers, cytoplasmic male-sterility (CMS)-based hybrid technology has been developed in pigeonpea (Saxena et al. 2010a). For instance, recently the ICPH 2671 hybrid developed using A_4_ cytoplasm has been released for commercial cultivation in India. The availability of a CMS system circumvents the need for manual emasculation and crossing, which is more suitable for commercial hybrid seed production. However, identification of a good restorer is cumbersome and time consuming as it requires extensive field evaluation.

Molecular breeding seems to be the next step for genetic improvement in pigeonpea. Molecular tools, such as DNA markers and genetic maps are essential prerequisites for undertaking any molecular breeding programme. Using these tools, QTLs or genes for traits of interest are identified and the markers linked with the QTLs/genes can be used to select the superior progenies in breeding programme. Among various kinds of markers systems available, simple sequence repeat (SSR) is preferred as the marker of choice for the plant breeding and genetics community (Gupta and Varshney 2000) and have been used successfully for genetic mapping and tagging of many agronomically important traits in several crop species. Advances in genomics, next generation sequencing (NGS) technologies and high-throughput (HTP) genotyping facilities, have provided automation-driven marker systems, such as single nucleotide polymorphism (SNP) markers. However, in the case of orphan legumes, such as pigeonpea, efforts are still underway to exploit the full potential of these technologies (Varshney et al. 2010a), while SSR markers have already proven of widespread value in molecular studies.

The low level of genetic diversity and less availability of DNA markers have hindered progress of development of saturated genetic maps in pigeonpea. Despite this, an SSR based genetic map derived from an inter-specific cross (*Cajanus cajan* × *C. scarabaeoides*) with moderate marker density has been reported in pigeonpea (Bohra et al. 2011). However, the genetic maps developed for cultivated pigeonpea so far (Gnanesh et al. 2011), are still suffering from the problem of poor map resolution due to the low polymorphism available between parental lines. For instance, the recently developed individual intra-specific genetic maps derived from the F_2_ populations viz. ICP 8863 × ICPL 20097 and TTB 7 × ICP 7035 have 120 and 78 SSR loci, respectively.

Considering the above, the construction of an integrated genetic map for cultivated pigeonpea offers a viable alternative to address the problem of low polymorphism through providing better genome coverage in comparison to population specific genetic maps. Apart from this, an integrated genetic map provides an excellent platform to target several important traits since individual mapping populations, may not segregate for many traits.

In this study, we report development of four genetic maps based on intra-specific F_2_ populations, of which three populations were segregating for fertility restoration. Subsequently, the first consensus genetic map after merging six SSR-based genetic maps has been developed. In addition, an attempt has been made to identify the genomic regions or QTLs associated with fertility restoration from three different genetic backgrounds.

## Materials and methods

### Mapping populations and DNA extraction

Four F_2_ mapping populations: ICPB 2049 × ICPL 99050, ICPA 2039 × ICPR 2447, ICPA 2043 × ICPR 3467 and ICPA 2043 × ICPR 2671 comprising of 188 individuals each, were used for construction of genetic maps. Phenotyping and QTL analysis for fertility restoration was done for the last three populations. The A-lines viz. ICPA 2039 and ICPA 2043, used in the three crosses were alloplasmic CMS lines based on A_4_ cytoplasm derived from wild progenitor *C. cajanifolius* (Saxena et al. 2010a). Genomic DNA from mapping parents and populations was isolated from leaf tissue and purified following Cuc et al. (2008).

### Phenotyping of mapping populations for pollen fertility

For assessing pollen fertility, 10 fully grown but un-opened floral buds were collected from different parts of the plants between 9 and 11 a.m. to prepare microscope glass slides for examination. Anthers from the sampled flowers were removed and squashed in 1 % aceto-carmine solution. In each glass slide, three different microscopic fields were studied under light microscope. The pollen grains were considered fertile if they were stained with dye and sterile if they were not stained (Gulyas et al. 2006). Within each population, discrimination among the plants for male-fertility restorers and non-restorers was done on the basis of their pollen fertility data. Plants with ≥80 % stained pollen grains were classified as male-fertile; while those with ≤10 % pollen fertility were identified as male-sterile.

### PCR and SSR analysis

Markers polymorphic between the parental lines as identified in Bohra et al. (2011) were used for genotyping the respective mapping population. Polymerase chain reactions (PCRs) for amplification of SSR loci were performed in a 384-well micro titre plate (ABgene, Rockford, IL, USA) using thermal cycler GeneAmp PCR System 9700 (Applied Biosystems, Foster City, CA, USA). The reaction volume consisted of 5 μl containing 0.5 μl of 10 × PCR buffer (SibEnzyme, Novosibirsk, Russia), 1.0 μl of 15 mM MgCl_2_, 0.25 μl of 2 mM dNTPs, 0.50 μl of 2 pmol/μl primer anchored with M13-tail (MWG-Biotech AG, Bangalore, India), 0.1 U of *Taq* polymerase (SibEnzyme, Novosibirsk, Russia) and 1.0 μl (5 ng/μl) of template DNA. A touch down PCR programme was used to amplify the DNA fragments: initial denaturation was for 5 min at 95 °C followed by five cycles of denaturation for 20 s at 94 °C, annealing for 20 s at 60 °C (the annealing temperature for each cycle being reduced by 1 °C per cycle) and extension for 30 s at 72 °C. Subsequently, 35 cycles of denaturation at 94 °C for 20 s followed by annealing for 20 s at 56 °C and extension for 30 s at 72 °C and 20 min of final extension at 72 °C. The PCR products were checked for amplification on 1.2 % agarose gel. Amplified products were separated on capillary electrophoresis using ABI 3730 (Applied Biosystems, Foster City, CA, USA) and allele calling was performed using GeneMapper software version 4.0 (Applied Biosystems, Foster City, CA, USA).

### Construction of component genetic maps

Genotype data were assembled for all segregating makers on all 188 F_2_ individuals from four mapping populations and linkage analysis was performed using JoinMap version 3.0 using “Regression mapping algorithm” (Van Ooijen and Voorrips 2001). Before linkage analysis, marker segregations in all populations were subjected to goodness of fit test to assess deviations from the expected Mendelian segregation ratio of 1:2:1 at 5 % level of significance. “Locus genotype frequency” function was used to calculate the χ^2^ values for all the markers. Map calculations were performed with parameters like LOD value ≥3.0, recombination frequency ≤0.40 and a χ^2^ jump threshold for removal of loci = 5. Addition of a new locus may influence the optimum map order; hence, a “Ripple” was performed after adding each marker into the map. Map distances were calculated using Kosambi mapping function (Kosambi 1944) and a third round was set to allow mapping of optimum number of loci in the genetic map. Placement of markers into different linkage groups (LGs) was done with “LOD groupings” and “Create group using the mapping tree” commands. Mean χ^2^ contributions or average contributions to the goodness of fit of each locus were also checked to determine the best fitting position for markers in genetic maps. The markers showing negative map distances or a large jump in mean χ^2^ values were discarded. Final maps were drawn with the help of MapChart version 2.2 (Voorrips 2002).

### Construction of consensus genetic map

Genotype data for six F_2_ mapping populations including four mapping populations in this study and two mapping populations reported earlier (Gnanesh et al. 2011) were used for developing a consensus genetic map using software JoinMap version 3.0. In this approach, segregation data from all mapping populations on all or some individuals are used to achieve a consensus order of loci to be used to develop the synthetic or integrated map (Wenzl et al. 2006). Map integration was accomplished by following three steps (Truco et al. 2007):

1. *A priori* identification of common loci among different mapping populations was carried out and their relative positions in different genetic maps were used to derive a consensus or framework order.
2. Finally “Combine groups for map integration” function from the “Join” menu was applied to synthesize an integrated LG.
3. The framework order of common markers obtained from step (1) was kept as *fixed* for map calculations of integrated LG using “Fixed order” command.

Problematic anchor loci in framework order, identified on the basis of mean χ^2^ statistics, were taken out from *fixed order*. To assess the amount of co-linearity in marker orders between consensus and component genetic maps, correlation coefficients (*r*) were calculated from marker positions in consensus and individual genetic map and their significance was tested. All the developed genetic maps were aligned together using a comparative mapping programme CMap version 1.01 to visually assess the congruency of marker orders.

### QTL analysis for fertility restoration

QTL analysis of fertility restoration from three mapping populations (ICPA 2039 × ICPR 2447, ICPA 2043 × ICPR 3467 and ICPA 2043 × ICPR 2671) were undertaken employing composite interval mapping (CIM) in the WinQTL Cartographer version 2.5 (Wang et al. 2007). CIM analysis was performed applying the Standard Model 6, with a genome scan interval (walk speed) of 1 cM. The “forward–backward stepwise regression” was used to set number of marker cofactors as background control. A window size of 10 cM was used to block out signals within 10 cM on either side of the flanking markers or QTL test site. Thresholds were determined by permutation tests using 1,000 permutations and a significance level of 0.05.

## Results

### Marker genotyping and segregation

Screening of 3,072 SSR markers on 22 parental genotypes of 13 mapping populations provided a set of 842 polymorphic markers which consisted of markers exhibiting polymorphism at least within one parental combination (Bohra et al. 2011). Based on the marker polymorphism data, a genetic map based on the inter-specific mapping population (*C. cajan* ICP 28 × *C. scarabaeoides* ICPW 94) with 239 SSR loci (Bohra et al. 2011) and two genetic maps based on the intra-specific mapping populations that segregate for sterility mosaic disease (SMD) viz. ICP 8863 × ICPL 20097 and TTB 7 × ICP 7035, with 120 and 78 SSR loci, respectively, were developed (Gnanesh et al. 2011). Genotyping of four new intra-specific mapping populations: ICPB 2049 × ICPL 99050, ICPA 2039 × ICPR 2447, ICPA 2043 × ICPR 3467 and ICPA 2043 × ICPR 2671 was done in this study using polymorphic SSR markers identified by Bohra et al. (ESM Table 1). These mapping populations segregate for different traits, such as *Fusarium* wilt (FW) (ICPB 2049 × ICPL 99050) and fertility restoration (Rf) (ICPA 2039 × ICPR 2447, ICPA 2043 × ICPR 3467 and ICPA 2043 × ICPR 2671) (Varshney et al. 2010b).

In summary, segregation data were assembled for 104, 83, 166 and 145 polymorphic markers on populations ICPB 2049 × ICPL 99050, ICPA 2039 × ICPR 2447, ICPA 2043 × ICPR 3467 and ICPA 2043 × ICPR 2671, respectively. Marker segregation data from each population was subjected to goodness of fit tests to assess the deviation from expected Mendelian ratio of 1:2:1 at the threshold of *p* = 0.05 (ESM Table 1, ESM Fig. 1).

### Component or individual genetic maps

Genotype data generated for all four intra-specific mapping populations were used to develop the components genetic maps for individual mapping populations. Two intra-specific genetic maps, reported earlier (Gnanesh et al. 2011), were also included for further analysis in this study. The percentage of markers, showing significant deviation from expected 1:2:1 ratio varied from 4.2 % (ICP 8863 × ICPL 20097) to 29.3 % (ICPA 2043 × ICPR 3467) (Table 1) in different populations. These distorted loci were scattered on all LGs, but LG02, LG03 and LG04 exhibited a higher proportion of distorted loci as compared to other LGs (ESM Fig. 1).

**Table 1:** Features of component genetic maps

Name of F_2_ mapping population	ICP 8863 × ICPL 20097	ICPA 2043 × ICPR 3467	ICPA 2043 × ICPR 2671	ICPA 2039 × ICPR 2447	TTB 7 × ICP 7035	ICPB 2049 × ICPL 99050
Number of total scored markers	143	166	145	83	84	104
Number of markers showing segregation distortion	5 (3.5 %)	54 (32.5 %)	32 (22 %)	23 (27.7 %)	7 (8.3 %)	39 (37.5 %)
Number of total mapped loci	120	140	111	78	78	59
Number of distorted loci	5 (4.2 %)	41 (29.3 %)	15 (13.5 %)	20 (25.6 %)	7 (9 %)	20 (33.9 %)
Total map length (cM)	534.9	881.6	678	570.5	467	586
Range of mapped loci	2 (LG01)–23 (LG09)	7 (LG11)–18 (LG01,02,04)	2 (LG03)–17 (LG09)	4 (LG07)–11 (LG04)	3 (LG07)–12 (LG01)	2 (LG12)–11 (LG09)
Range of map lengths	6.8 (LG07)–105.9 (LG02)	49 (LG03)–125.9 (LG09)	22.5 (LG03)–139.4 (LG09)	9.0 (LG08)–118.7 (LG02)	4.3 (LG07)–89.5 (LG01)	19.3 (LG12)–112.9 (LG09)
Average inter-marker distance (cM)	4.5	6.3	6.1	7.3	6	9.9

In summary, the number of mapped loci across all the six intra-specific genetic maps ranged from 59 (ICPB 2049 × ICPL 99050) to 140 (ICPA 2043 × ICPR 3467) (Table 1). In all the genetic maps, 11 linkage groups (LGs) were obtained except for population ICPB 2049 × ICPL 99050 with 12 LGs. The maximum map length was shown by ICPA 2043 × ICPR 3467 (881.6 cM) genetic map while minimum of 467 cM was observed for TTB 7 × ICP 7035. Average inter-marker distance varied from 4.5 cM (ICP 8863 × ICPL 20097) to 9.9 cM (ICPB 2049 × ICPL 99050) (http://www.cmap.icrisat.ac.in/cmap/sm/pp/bohra/).

### The consensus genetic map

The availability of a sufficient number of common markers on six intra-specific genetic maps facilitated the merging of six maps into one consensus map. While integrating different genetic maps, the nomenclature of common markers present on component genetic maps is crucial (Varshney et al. 2007). In the present study, however, there was no discrepancy in names of common markers, since 98.8 % of the markers used for linkage analysis came from the same source, i.e. BAC-end derived SSRs and designated as *Cajanus* *cajan* microsatellite (CcM) markers. Segregation data for 348 markers obtained on 6 different mapping populations was used for merging multiple genetic maps. Although 203 markers were unique to individual genetic maps, 145 markers were common among two (80 markers), three (43 markers), four (16 markers) and five (6 markers) mapping populations that served as anchor points for map integration (Table 2). Most of the LGs of component populations were successfully integrated into the consensus map. Details of the consensus map and markers contributed from different component genetic maps have been given in Table 3.

**Table 2:** Number of common markers among different component mapping populations

S. no.	Mapping populations	Number of F_2_ lines	Total number of mapped markers	Number of markers common to ‘*n*’ number of mapping populations				
				*n* = 0	*n* = 1	*n* = 2	*n* = 3	*n* = 4
1	ICP 8863 × ICPL 20097	190	120	61	22	20	13	4
2	ICPA 2043 × ICPR 3467	188	140	36	49	35	15	5
3	ICPA 2043 × ICPR 2671	188	111	19	43	29	14	6
4	ICPA 2039 × ICPR 2447	188	78	26	15	22	9	6
5	TTB 7 × ICP 7035	130	78	33	16	15	9	5
6	ICPB 2049 × ICPL 99050	188	59	28	15	8	4	4
	Total			203	80	43	16	6

**Table 3:** Summary of consensus genetic map

Consensus map				Number of the markers contributed from component genetic maps					
Linkage groups	Number of markers^a^	Map distance (cM)	Average inter-marker distance (cM)	ICP 8863 × ICPL 20097	ICPA 2043 × ICPR 3467	ICPA 2043 × ICPR 2671	ICPA 2039 × ICPR 2447	TTB 7 × ICP 7035	ICPB 2049 × ICPL 99050
1	32 (14)	99.9	3.1	–	17	9	9	12	1
2	39 (19)	135.2	3.5	13	19	13	10	7	8
3	29 (9)	83.7	2.9	11	13	3	8	6	1
4	46 (16)	83.9	1.8	15	18	16	11	7	13 (LG04 + LG05)
5	23 (12)	83.8	3.6	9	10	10	5	8	–
6	50 (17)	78.0	1.6	21	15	15	8	7	10 (LG06 + LG08)
7	18 (9)	84.9	4.7	5	8	10	4	3	4
8	16 (9)	57.5	3.6	8	8	7	1	6	–
9	45 (24)	128.0	2.8	22	17	17	6	8	13 (LG09 + LG10)
10	30 (12)	101.0	3.4	12	9	10	8	8	–
11	11 (6)	123.1	11.2	–	7	3	6	1	–
Total	339	99.9	3.1	116	141	113	76	73	50

^a^Number given in parenthesis indicates the common markers existing in that LG

**Fig. 1:**
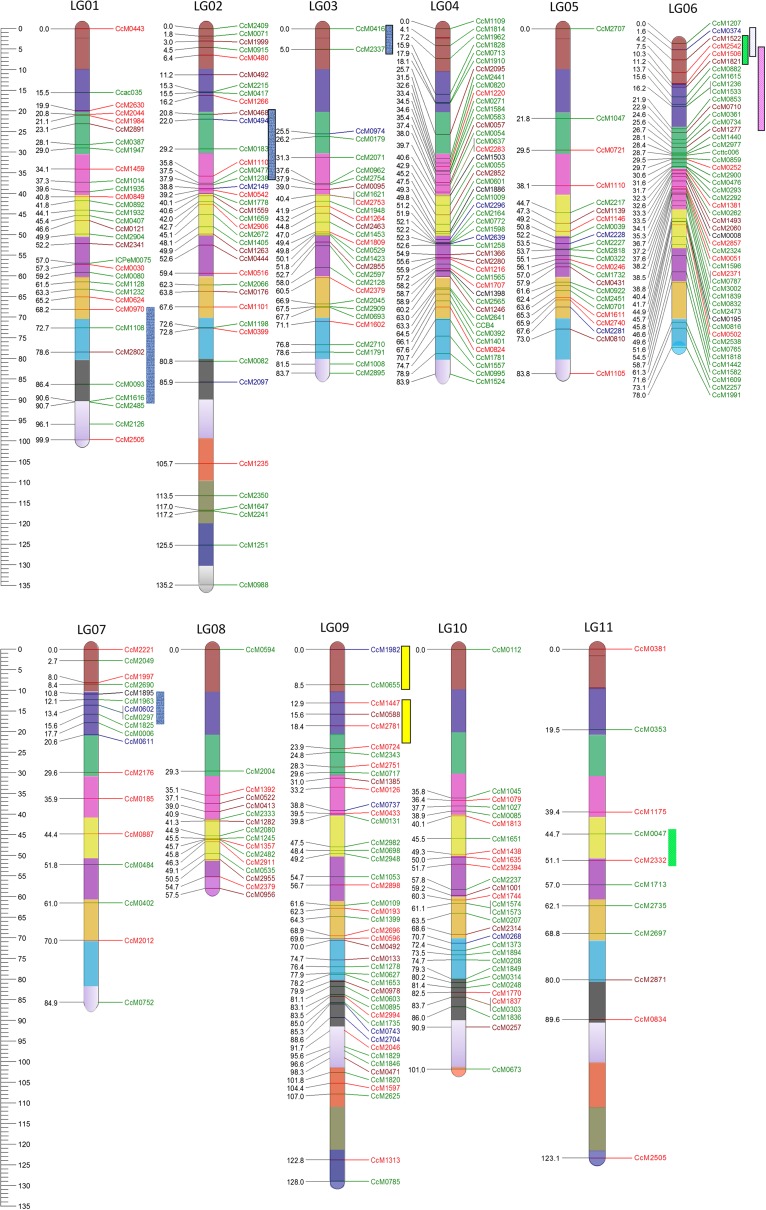
A consensus genetic map comprising 339 loci. Markers are shown on *right* *side* of the LG while map distances are indicated on *left* *side*. Each LG is divided into several bins based on 10-cM interval. The markers unique to mapping populations, common between two, three, four and five mapping populations have been shown by *green*, *red*, *brown*, *blue* and *black* *colour*, respectively. QTLs are indicated by *bars* with *different colours*. *Blue*, *green*, *pink*, *white* and *yellow coloured bars* were used to show the QTLs derived from populations TTB 7 × ICP 7035, ICPA 2039 × ICPR 2447, ICPA 2043 × ICPR 2671, ICPA 2043 × ICPR 3467 and ICP 8863 × ICPL 20097, respectively

All the common markers collectively led to the synthesis of a consensus map comprising 339 loci on 11 LGs and covering a map distance of 1,059 cM (Fig. 1; Table 3). In the consensus map, a total of 147 (43.4 %) markers were anchor markers and the percentage of these markers varied from 31.0 % (LG03) to 54 % (LG11) across different LGs. The remaining 192 (56.6 %) markers in the consensus map were unique to individual mapping populations. It is important to note that four markers namely CcM0492 (mapped on LG02 and LG09), CcM1110 (mapped on LG02 and LG05), CcM2379 (mapped on LG03 and LG08) and CcM2505 (mapped on LG01 and LG11) were mapped on different LGs in different crosses. Two of the anchor markers couldn’t integrate into consensus map and another four were mapped at two different loci hence the total number becomes 147 instead of 145.

The number of markers per linkage group on the consensus map varied form 11 (LG11) to 50 (LG06). The LG02 exhibited maximum map length of 135.2 cM while minimum map length (57.5 cM) was observed for the LG08. The average inter-marker distance ranged from 1.6 cM (LG06) to 11.2 cM (LG11) with an average of 3.1 cM. Non-uniform distribution of markers was evident in all LGs. Visual inspection of the consensus map resulted in identification of only 15 major gaps (> 10 cM) across all the LGs except for LG04 which did not show any major gap. The largest gap between two loci was found to be 35.8 cM between markers CcM0112 (at 0 cM) and CcM1045 (at 37.8 cM) on LG10 followed by 33.5 cM between CcM0834 (at 89.6 cM) and CcM2505 (at 123.1 cM) on LG11 (Fig. 1, ESM Fig. 1, http://www.cmap.icrisat.ac.in/cmap/sm/pp/bohra/).

In terms of SSR motifs, the majority (55.45 %) of the markers integrated into the consensus map, belonged to the di-nucleotide repeat category followed by compound type SSRs (28.90 %) (ESM Table 2). The lowest representation was from tetra and hexa-nucleotide repeat classes. More than 58 % of the markers in the consensus map exhibited polymorphism information content (PIC) values greater than 0.5, with 28 % having PIC values greater than 0.75. Average PIC value of individual LGs varied from 0.64 (LG04) to 0.72 (LG11) while average number of alleles ranged form 6.5 (LG08) to 7.2 (LG10). The consensus map was divided into several bins of 10 cM each to aid future genetic mapping and diversity analysis (Fig. 1, ESM Fig. 1). As expected, the SSR markers present in each bin have varied PIC values (ESM Table 2). Now the community can select the highly informative SSR markers from each bin that will best represent the genome in the germplasm to be analyzed.

With the objective to make the consensus map more informative, QTLs for fertility restoration identified in this study and for SMD resistance based on two mapping populations (TTB 7 × ICP 7035 and ICP 8863 × ICPL 20097) identified by Gnanesh and colleagues, were placed on the consensus map (Fig. 1). Placement of all these QTLs into a single genetic map will facilitate the adoption of the identified QTLs for SMD resistance and fertility restoration in pigeonpea breeding. For instance, a QTL associated with SMD resistance namely qSMD3, bracketed by markers CcM2149 (PIC value: 0.73) and CcM0468 (PIC value: 0.67), was identified on LG02 from one of the component population TTB 7 × ICP 7035 (Gnanesh et al. 2011). In the consensus map, five additional markers namely CcM0494, CcM0183, CcM1110, CcM0477 and CcM1238 were integrated into this QTL region. Among these new markers, CcM0494 and CcM0183 with PIC values of 0.86 and 0.78 respectively as compared to CcM2149 and CcM0468 identified originally, will be more valuable while screening the germplasm for resistance to SMD. Similarly, localization of all the three RF-QTL regions, identified on LG06, into a single genetic map provided a common region i.e. marker interval CcM2842–CcM1506, that may be associated with fertility restoration in all three genetic backgrounds.

### Comparison of consensus map and component maps

**Fig. 2:**
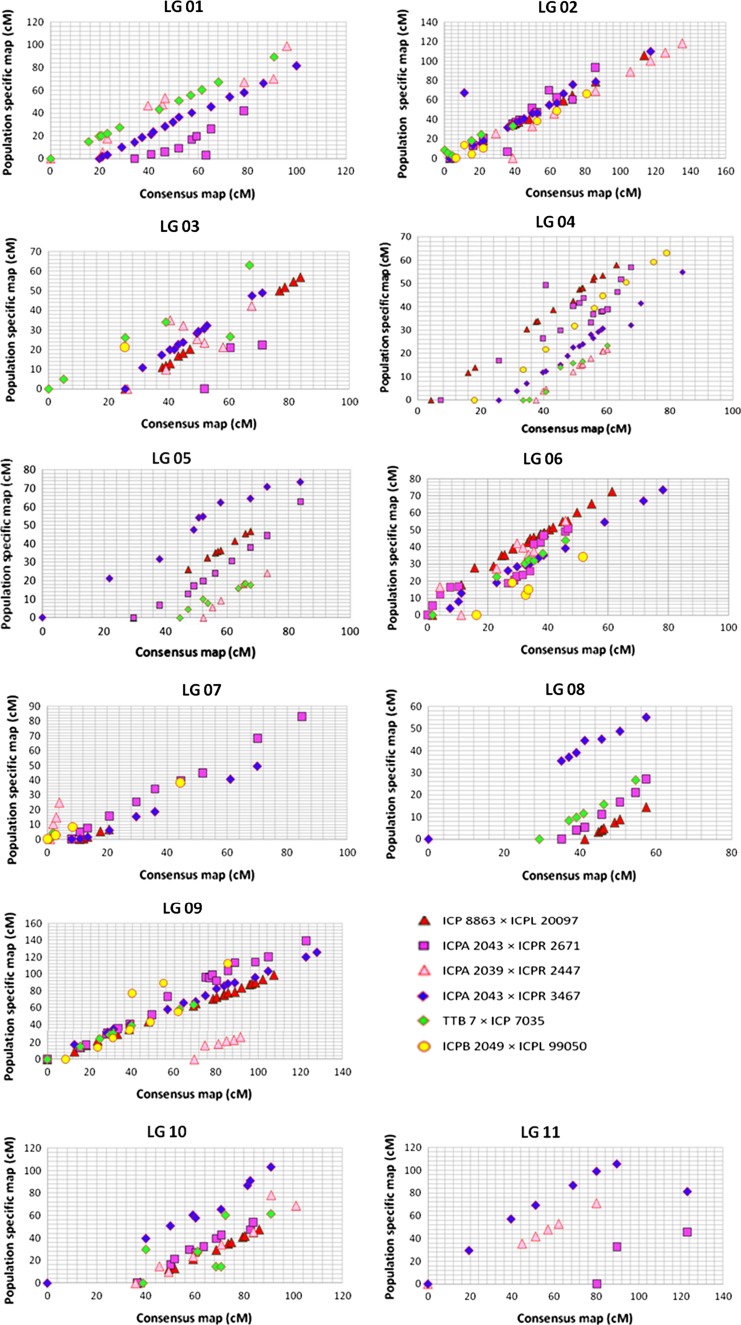
Scatter plots showing the extent of correlations among consensus genetic map and population-specific genetic maps. The marker integrated from different populations viz. ICP 8863 × ICPL 20097, ICPA 2039 × ICPR 2447, ICPA 2043 × ICPR 2671, ICPA 2043 × ICPR 3467, TTB 7 × ICP 7035 and ICPB 2049 × ICPL 99050 are shown by *red triangles*, *pink triangles*, *purple squares*, *blue diamonds*, *light*-*green diamonds* and *yellow circles*, respectively

**Fig. 3:**
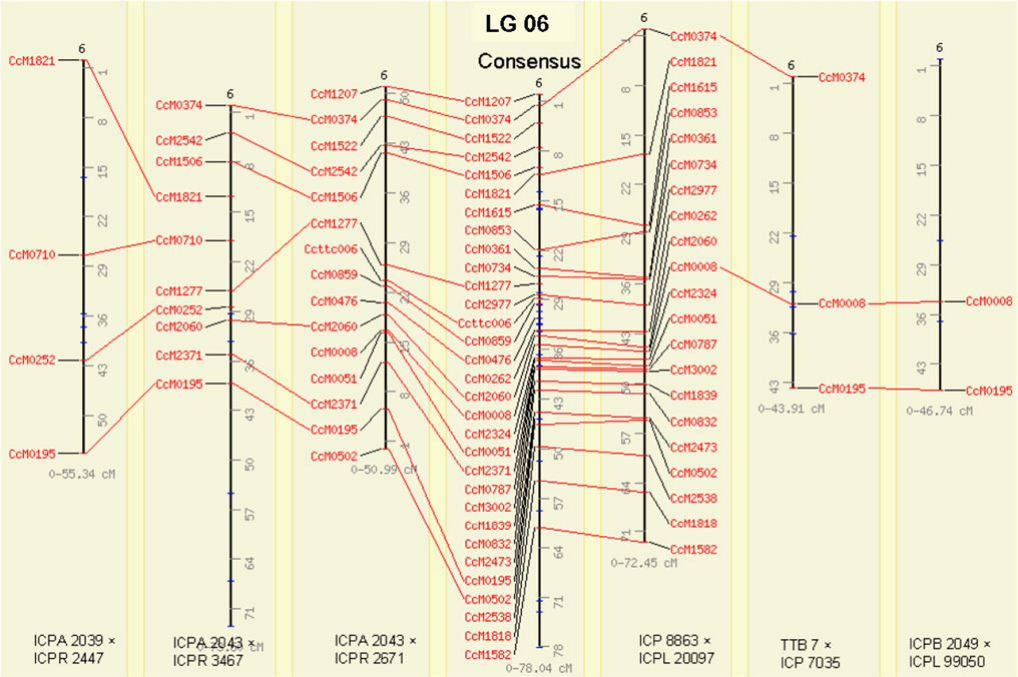
This depicts the marker-based correspondences for LG06, among consensus and individual genetic maps. Only common markers i.e. landmarks are included to visually asses the co-linearity of marker orders and marker positions. LGs are aligned together using comparative mapping programme CMap version 1.01. Figure can also be found at http://www.cmap.icrisat.ac.in/cmap/sm/pp/bohra/

Nomenclature of LGs in the consensus as well as in component genetic maps were given according to the reference genetic map of pigeonpea derived from an inter-specific F_2_ (ICP 28 × ICPW 94) population. Detailed comparison of the consensus map and population-specific genetic maps has revealed a very high degree of conservation in marker orders and marker groupings. For instance, a high degree of correlation (correlation coefficients varying from 0.64 to 0.99) was observed for all the LGs between consensus and population specific LGs. The highest amount of co-linearity with the consensus map was exhibited by the ICP 8863 × ICPL 20097 genetic map, which consistently showed correlation coefficients of 0.99 for the nine linkage groups merged into consensus map. Highly significant values of correlation coefficients showed a good agreement of both marker orders and markers positions or inter-marker distances between consensus and component genetic maps (Fig. 2). As an example, comparison of LG06 for all the maps using CMap version 1.01 has been shown in Fig. 3. A detailed comparison of all linkage groups across all the maps has been shown in ESM Fig. 2. CMap helps in assessing the congruency of marker positions and orders by making a pairwise comparison between different genetic maps. Considering only the common loci existing among various genetic maps, highly conserved marker orders were manifested.

### Comparison of consensus map with the inter-specific genetic map

**Fig. 4:**
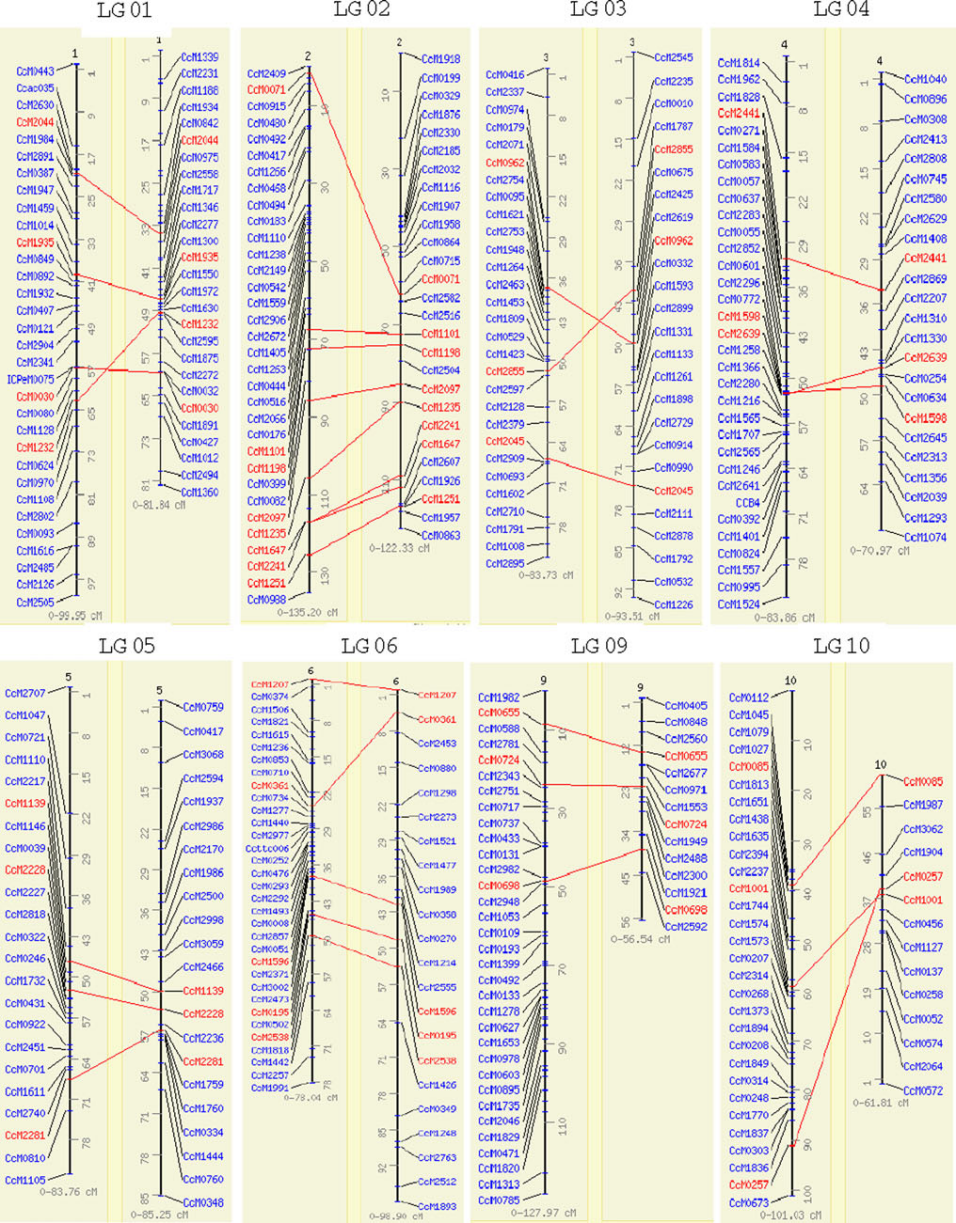
Comparison of marker order between the consensus and inter-specific genetic map based on ICP 28 × ICPW 94 mapping population. Consensus LGs are on *left side* while inter-specific LGs are on *right side*. Common loci are indicated by *red colour*, while unique loci are shown by *blue colour*

With the objective of assessing the consistency of marker orders and possible rearrangements between the intra-specific and inter-specific genetic maps, the consensus map (339 SSR loci) developed in this study was compared with a reference genetic map (ICP 28 × ICPW 94) developed by Bohra and colleagues (Fig. 4). Between these maps, a total of 38 markers were common and scattered on all 11 linkage groups. Out of these 38 common markers, six markers; namely, CcM2911, CcM0417, CcM0392, CcM1781, CcM0603 and CcM0752 had different positions. Three of these makers had significant segregation distortion (CcM2911: χ^2^ = 17.3, CcM0417: χ^2^ = 17.4. and CcM0392: χ^2^ = 78.5) in the inter-specific cross. Each of the six markers was mapped only in one of the six intra-specific mapping populations and therefore these markers were not used as anchor markers. Nevertheless, these markers were included in the consensus genetic map. However, some inconsistency was observed in the genetic mapping positions for these markers between the consensus map and the inter-specific genetic map that may be the result of mapping of two different loci/fragments in the inter-specific and intra-specific mapping populations.

The remaining 32 markers were mapped to the same position on the LGs in both consensus map and inter-specific genetic maps. Marker positions were found to be fairly concurrent between these two genetic maps. Although five markers (CcM1232, CcM1647, CcM2855, CcM2639 and CcM0257) showed slight difference in their position along LG, most of these were consecutive pairs, so still found on the same genomic regions.

### Phenotyping and QTLs for fertility restoration

Three of the mapping populations used in this study segregate for fertility restoration (ICPA 2039 × ICPR 2447, ICPA 2043 × ICPR 3467 and ICPA 2043 × ICPR 2671) and were phenotyped for fertility restoration. The cross ICPA 2039 × ICPR 2447 belonged to the early maturing category while the latter two crosses were from the late maturing category. In all the crosses, fully fertile F_1_s with good pollen load were recovered indicating dominant nature of loci involved in fertility restoration. In the F_2_, the phenotypic segregation for fertility restoration was observed and data were recorded on 188 individuals of each the three crosses (Table 4).

**Table 4:** Descriptive statistics of phenotyping data on fertility restoration

Mapping populations	Sample size	Mean	Min.	Max.	Standard deviation	Standard error	Skewness	Kurtosis
ICPA 2039 × ICPR 2447	188	86.86	0.0	100	22.70	1.65	−3.42	10.24
ICPA 2043 × ICPR 2671	188	89.11	5.6	100	16.94	1.23	−4.19	17.22
ICPA 2043 × ICPR 3467	188	81.34	1.6	100	30.14	2.19	−2.13	2.68

QTL mapping for fertility restoration was done based on arc sine transformed values of mean phenotypic data of  percentage pollen fertility and genetic mapping data using CIM approach. CIM analysis revealed occurrence of a total of four major QTLs for fertility restoration across three different pedigrees (Table 5). These QTLs were designated as QTL-RF-1 to QTL-RF-4. Of the total QTLs identified, two QTLs namely QTL-RF-1 (flanked by CcM1821 and CcM1522) and QTL-RF-2 (flanked by CcM0047 and CcM2332) explaining 14.85 %, and 15.84 % of the PV respectively, were identified in ICPA 2039 × ICPR 2447 population. Similarly one major QTL viz. QTL-RF-3 (bracketed in CcM1277-CcM2542) explaining 20.89 % of PV was recovered from population ICPA 2043 × ICPR 2671. QTL analysis conducted on population I CPA 2043 × ICPR 3467 identified a single major QTL named as QTL-RF-4 (bracketed in CcM0374–CcM1506 region). This QTL contributing up to 24.17 % of PV was identified at a LOD value of 8.9. In terms of localization of RF-QTLs in linkage groups, the LG06 contained three QTLs (QTL-RF-1, QTL-RF-3 and QTL-RF-4) while the remaining single QTL viz. QTL-RF-2 was located on the LG11.

**Table 5:** Identification of QTLs for fertility restoration using CIM analysis

Mapping populations	Name of QTLs	Linkage group	Position (cM)	LOD	Flanking markers	*R* ^2^ or phenotypic variation (PV) (%)
ICPA 2039 × ICPR 2447	QTL-RF-1	LG06	8.0	3.9	CcM1522–CcM1821	14.85
	QTL-RF-2	LG11	36.4	5.3	CcM0047–CcM2332	15.84
ICPA 2043 × ICPR 2671	QTL-RF-3	LG06	35.8	4.8	CcM2542–CcM1277	20.89
ICPA 2043 × ICPR 3467	QTL-RF-4	LG06	3.9	8.9	CcM0374–CcM1506	24.17

## Discussion

Molecular markers and genetic maps are prerequisites for undertaking trait mapping and molecular breeding in any crop species. While significant progress has been made in cereals (Varshney et al. 2005) and a few legume species (Varshney et al. 2010c), in the case of pigeonpea, because of its narrow genetic base, together with the paucity of molecular markers and mapping populations, the crop did not have a genetic map until 2010 (Varshney et al. 2010b). Only recently, a set of 3,200 SSR markers and an inter-specific reference genetic map have become available (Bohra et al. 2011). However, as for breeding applications, intra-specific genetic maps are more useful, only two intra-specific genetic maps with few QTLs for SMD have been reported so far (Gnanesh et al. 2011). The present study focuses on construction of four genetic maps based on intra-specific mapping populations of which three populations segregate for fertility restoration. These maps contain only 78 (ICPA 2039 × ICPR 2447) to 140 (ICPA 2043 × ICPR 3467) SSR loci even after scanning 3,200 SSR markers on the parental genotypes of the mapping populations. This low level of polymorphism and the low-density genetic maps have been reported earlier and the intra-specific genetic maps contained 78 (TTB 7 × ICP 7035) and 120 (ICP 8863 × ICPL 20097) SSR loci respectively (Gnanesh et al. 2011).

Segregation distortion was observed in all the six intra-specific crosses with varying degree of deviation. Segregation distortion is a common phenomenon observed in intra as well as in inter-specific crosses, however the extent is more in case of inter-specific crosses. For instance, percentage of distorted markers ranged from 3.49 % (ICP 8863 × ICPL 20097) to 37.50 % (ICPB 2049 × ICPL 99050) in intra-specific crosses, about 63.5 % SSR showed segregation distortion in inter-specific cross (Bohra et al. 2011). Similar instances of segregation distortion were also reported for *Medicago* (Jenczewski et al. 1997), chickpea (Gaur et al. 2011) and mungbean (Lambrides et al. 2000). Some of the regions on LG02, LG03 and LG04 (in the crosses ICPA 2039 × ICPR 2447, ICPA 2043 × ICPR 3467 and ICPB 2049 × ICPL 99050) can be considered as “segments associated with skewed segregation” because these regions harboured four or more closely linked markers showing significant and consistent deviation from expected F_2_ ratio of 1:2:1 (Xu et al. 1997; Marcel et al. 2007). Segregation distortion may result from various factors such as residual heterozygosity, gametic or zygotic selections and genotyping errors (Liang et al. 2006).

The prime objective of this study was to construct a high density integrated genetic map from different pedigrees with highly conserved marker orders that can be used as reference genetic map for cultivated crosses. As a result, we present the first integrated genetic map for cultivated pigeonpea that may be regarded as a “consensus map” as suggested by Isobe et al. (2009). The good agreement of marker orders as well as inter-marker distances observed among different component genetic maps may be due to (1) fairly similar population size (~188), (2) type of mapping populations (all F_2_s) and (3) type of marker system (co-dominant), taken into consideration for linkage analysis. Such consensus maps were developed earlier in many plant species like wheat (Somers et al. 2004), barley (Varshney et al. 2007; Marcel et al. 2007), red clover (Isobe et al. 2009), sorghum (Mace et al. 2009), soybean (Hyten et al. 2010), groundnut (Hong et al. 2010) and chickpea (Radhika et al. 2007; Millan et al. 2010). Consensus genetic maps, consolidating genetic information contained in different genetic backgrounds, offer a valuable resource for genetic analysis and breeding.

The average marker density (3.1 cM) in the consensus map is higher than recorded for inter-specific genetic map (3.8 cM) (*t* = 2.1 and *p* = 0.03) (Bohra et al. 2011). However, the slight difference in marker order relative to inter-specific genetic map may be accounted to genotyping errors. Secondly, all of these markers are located on the same genomic regions and flipping is a common phenomenon for closely spaced markers (Feltus et al. 2006; Wu and Huang 2006) which may be accounted to genotyping imprecision rather than real rearrangements (Lombard and Delourme 2001). Similar findings were also observed by Winter et al. (1999) and Millan et al. (2010) while comparing intra- and inter-specific genetic maps in chickpea. Poor correlation observed between length of LGs and number of markers/LG in consensus genetic map suggested non-uniform distribution of markers along LGs. This non-uniform distribution is mainly because of the gaps existing in distal ends of LGs which may be due to deficiency of markers in these regions (Sewell et al. 1999).

Most of the markers integrated into the consensus map were highly informative since more than 50 % of the markers exhibited PIC values greater than 0.50. Similarly, the average number of alleles (6.27) and average PIC value (0.67) of all mapped markers were higher than reported earlier (Burns et al. 2001; Odeny et al. 2007; Saxena et al. 2010b). The bin-wise information on PIC values provided for all integrated markers will help geneticists and breeders to select a good set of markers that will represent the genome as well as display high degree of polymorphism and such a set of markers will be very useful for developing new genetic maps, trait mapping and diversity analysis.

Marker-trait association analysis in three mapping populations provided the candidate molecular markers and QTLs for fertility restoration in hybrid breeding of pigeonpea. All four QTLs detected for fertility restoration contributed more than 10 % of phenotypic variation and these QTLs, therefore, can be considered as QTLs playing major roles in restoring fertility in A_4_ cytoplasm in pigeonpea. The fertility restoration has been subjected to QTL analyses in F_2_ population of several other crop species where CMS systems are well established such as wheat (Zhou et al. 2005), rice (Tan et al. 1998), pepper (Wang et al. 2004) etc. These studies reported existence of large effect QTLs governing major proportions of the phenotypic variation. However, presence of minor QTLs/genes was also observed which can act as modifiers in restoring the fertility and hence increasing complexity in fertility restoration phenomenon.

Moreover, the QTL region flanked by the markers CcM1506 and CcM2542 were found in two different genetic backgrounds. This indicates the utility of these common markers and consistent QTLs for hybrid breeding in pigeonpea. It is interesting to note that majority of the QTLs identified were located on the LG06 in all the three mapping populations indicating the underlying importance of the LG06. This is the first study on the identification of QTLs for fertility restoration in pigeonpea. Identification of SSR markers tightly linked with fertility restoration will assist pigeonpea breeders in quick discrimination between maintainer (B-lines) and restorer lines (R-lines). Since the absence of fertility restorer in B- line is an essential prerequisite for maintenance of sterile lines (A-lines). Furthermore, recovery of a potential restorer for CMS based hybrid development is very labour intensive and cumbersome procedure as it requires extensive test crossing and field screening to assess the level of fertility restoration through various A × R combinations (Yue et al. 2010). Furthermore, identification of good R-lines cannot be done before onset of flowering in A × R progenies. Hence, SSR marker would facilitate not only rapid selection of restorer lines but also ensure precise introgression of fertility restorer loci into elite pigeonpea breeding lines. Apart from QTLs governing fertility restoration, QTLs imparting SMD resistance were also placed in the consensus genetic map which allowed integration of more informative markers into QTL harbouring regions. Inclusion of additional markers in the QTL regions of the consensus genetic map provides an opportunity for selecting reliable markers from the region together with allowing comparison of the region of interest in different pedigrees.

In summary, four new intra-specific genetic maps have been constructed based on BAC end sequence (BES) derived SSR markers. All these genetic maps together with the two intra-specific genetic maps reported in earlier study, allowed development of a consensus genetic map comprising 339 loci with an average marker density of 3.1 cM. This is the *first* instance of integrating multiple component genetic maps in pigeonpea. Furthermore, grouping of markers into bins and associating them with PIC values on the integrated genetic map will facilitate the selection of evenly distributed markers for various genetics and breeding studies including genetic mapping (for new populations), association or linkage disequilibrium (LD) studies, diversity analysis, or for practicing background selection in molecular breeding studies aimed at crop improvement in pigeonpea. In parallel, QTL analysis performed on fertility restoration data, detected a total of four major QTLs, representing this study as a pioneering step towards molecular dissection of fertility restoration in pigeonpea. The identification of major RF-QTLs would open new avenues for genomics-assisted hybrid breeding in pigeonpea.

## Electronic supplementary material

Below is the link to the electronic supplementary material.

Supplementary material 1 (PPT 1768 kb)

Supplementary material 2 (PPT 628 kb)

Supplementary material 3 (XLS 1541 kb)

Supplementary material 4 (XLS 113 kb)
