# Supplementary material for: An intra-specific consensus genetic map of pigeonpea [Cajanus cajan (L.) Millspaugh] derived from six mapping populations
Source: Theor Appl Genet. 2012 Jul 8;125(6):1325–38. doi: 10.1007/s00122-012-1916-5 (PMC3442162; doi:10.1007/s00122-012-1916-5)
Supplement: Supplementary file 1 — Supplementary material 1 (PPT 1768 kb) [file 122_2012_1916_MOESM1_ESM.ppt]

## Slide 1
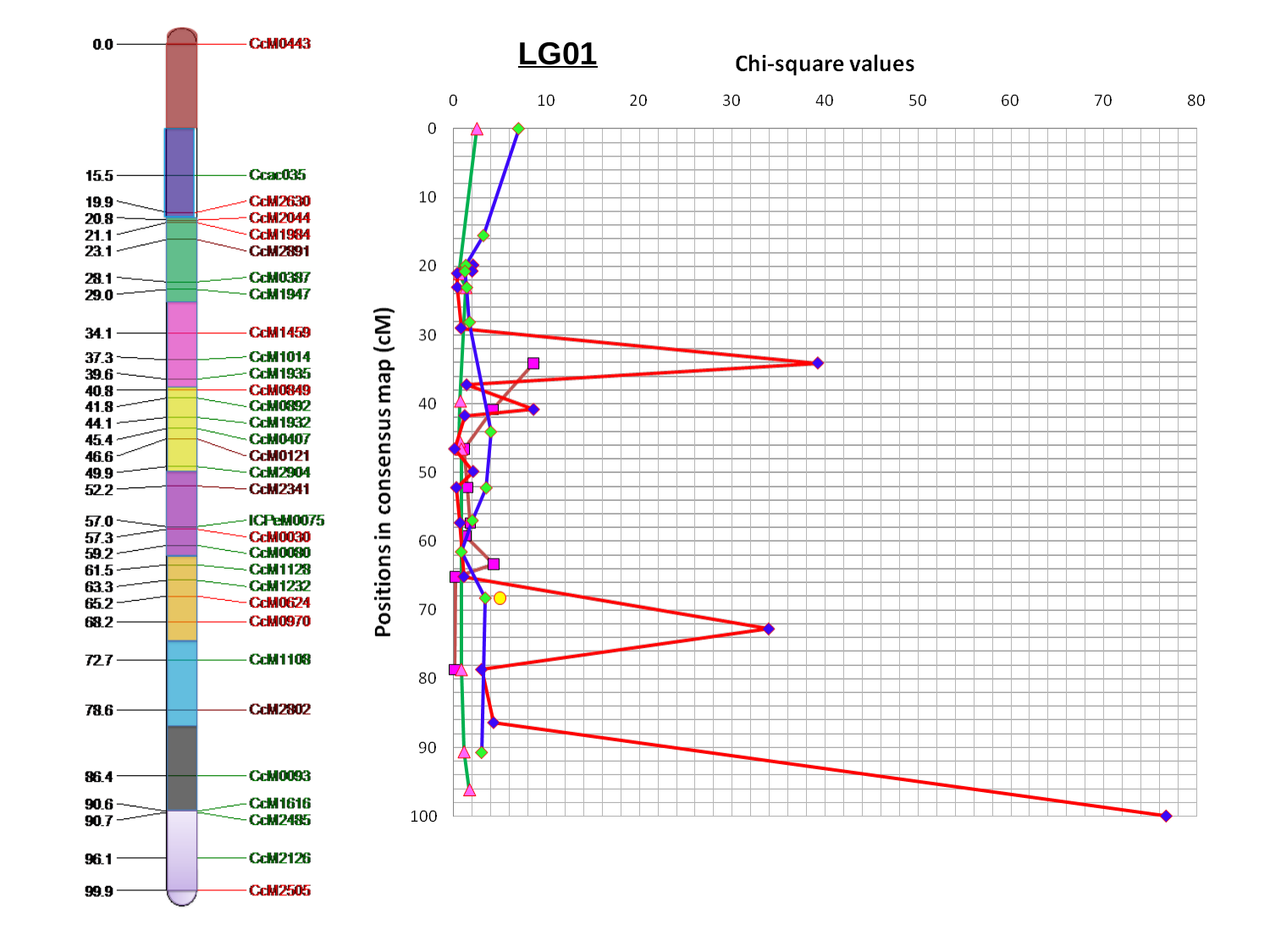

LG01

## Slide 2
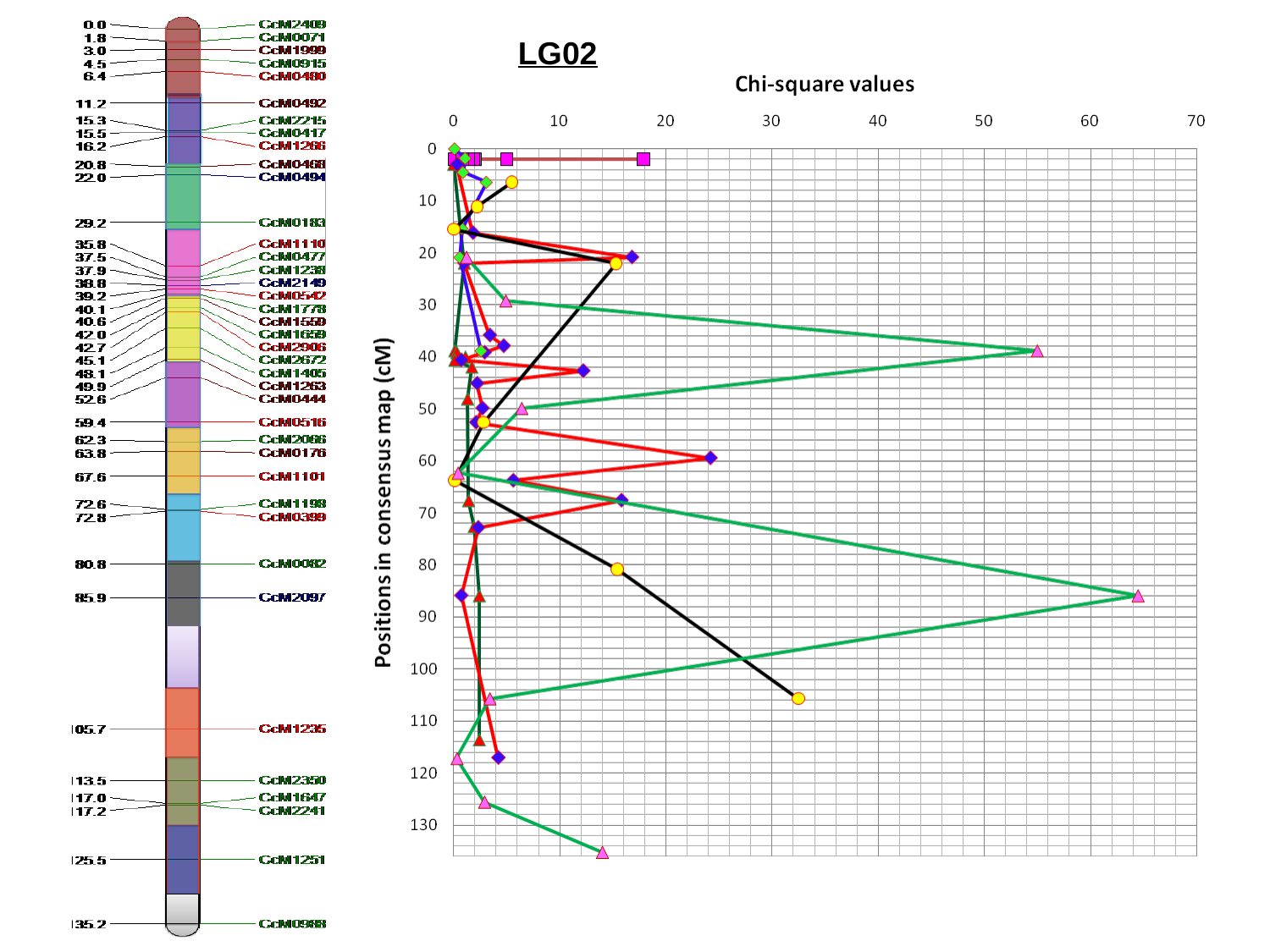

LG02

## Slide 3
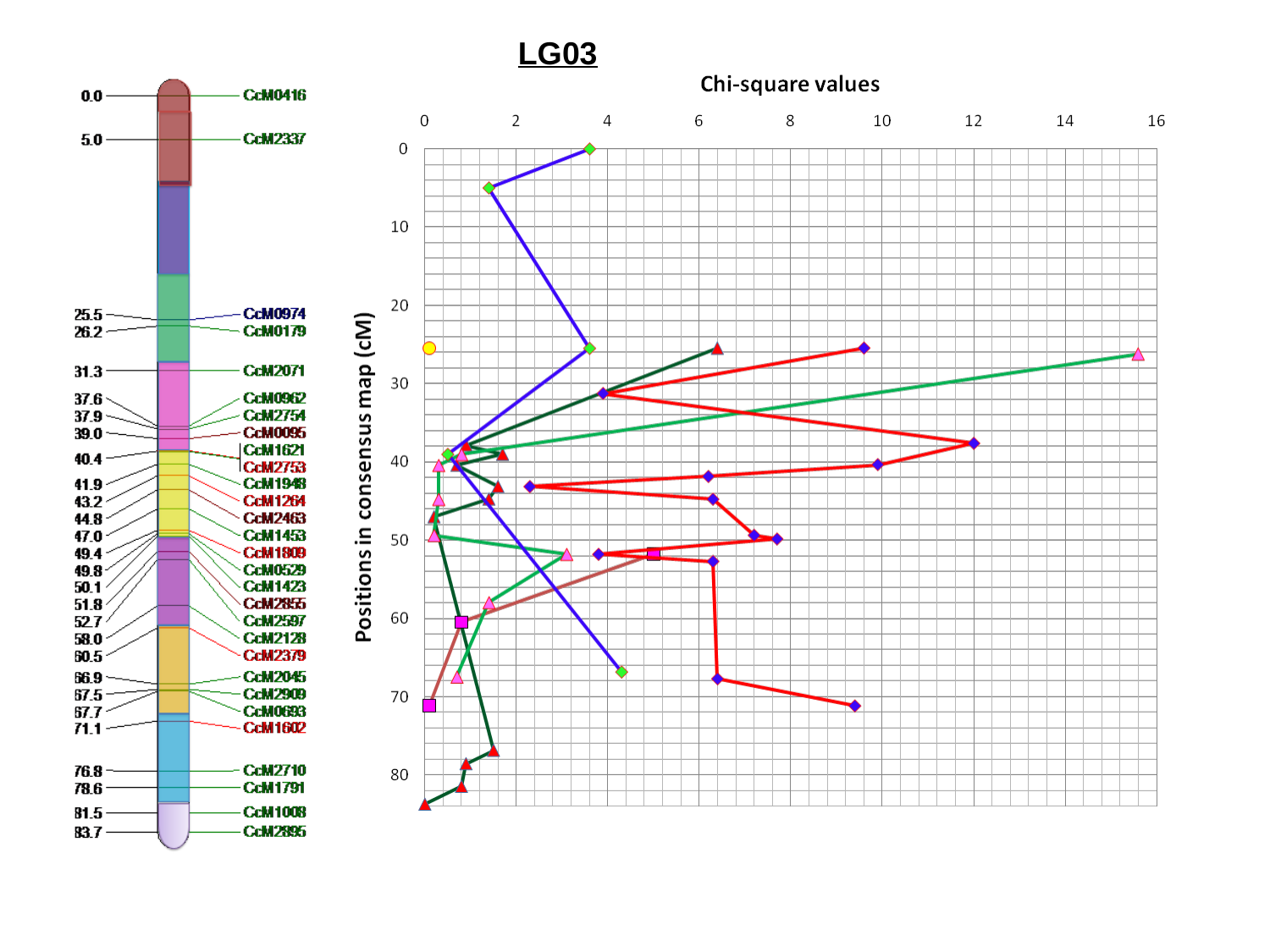

LG03

## Slide 4
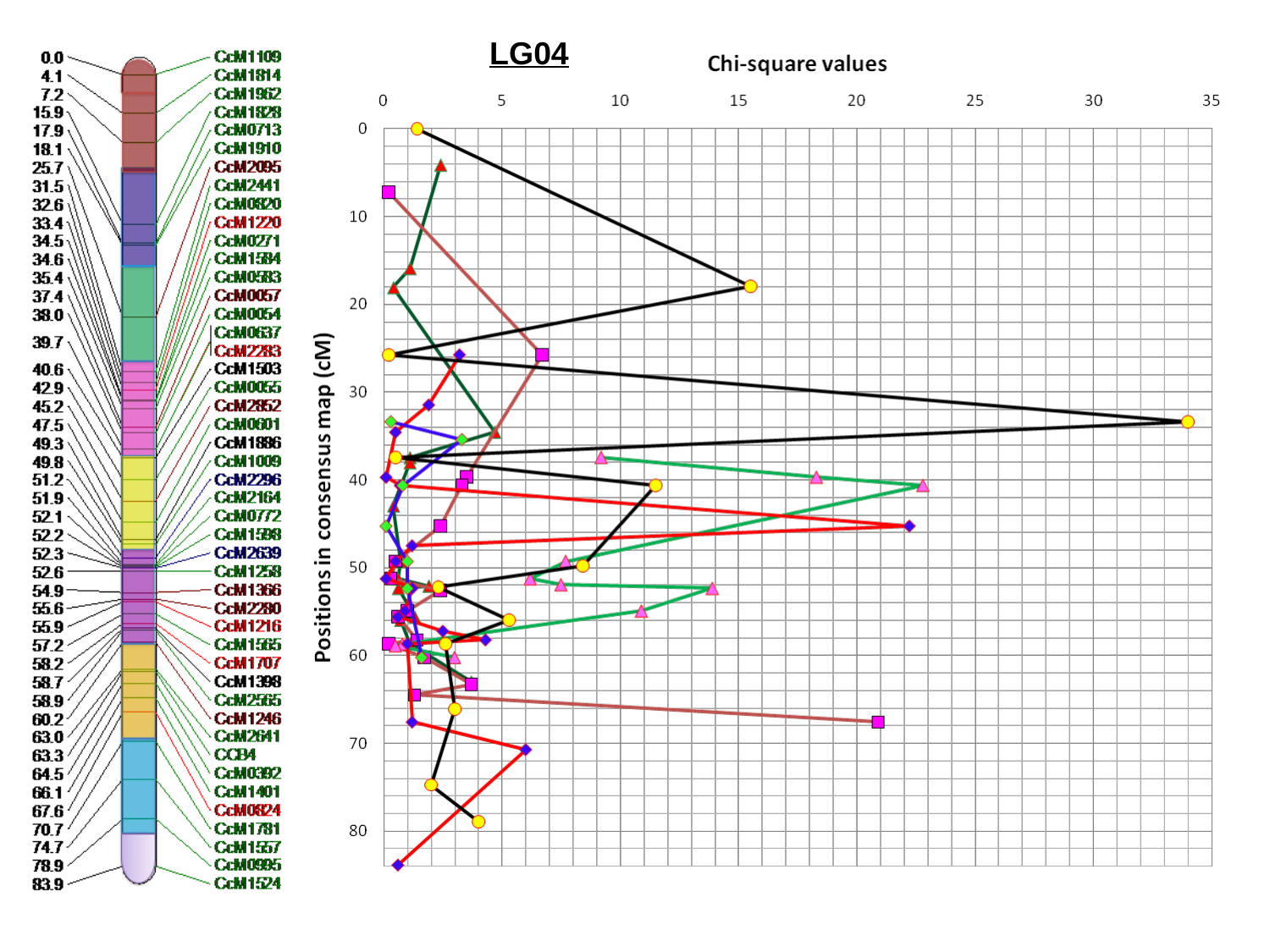

LG04

## Slide 5
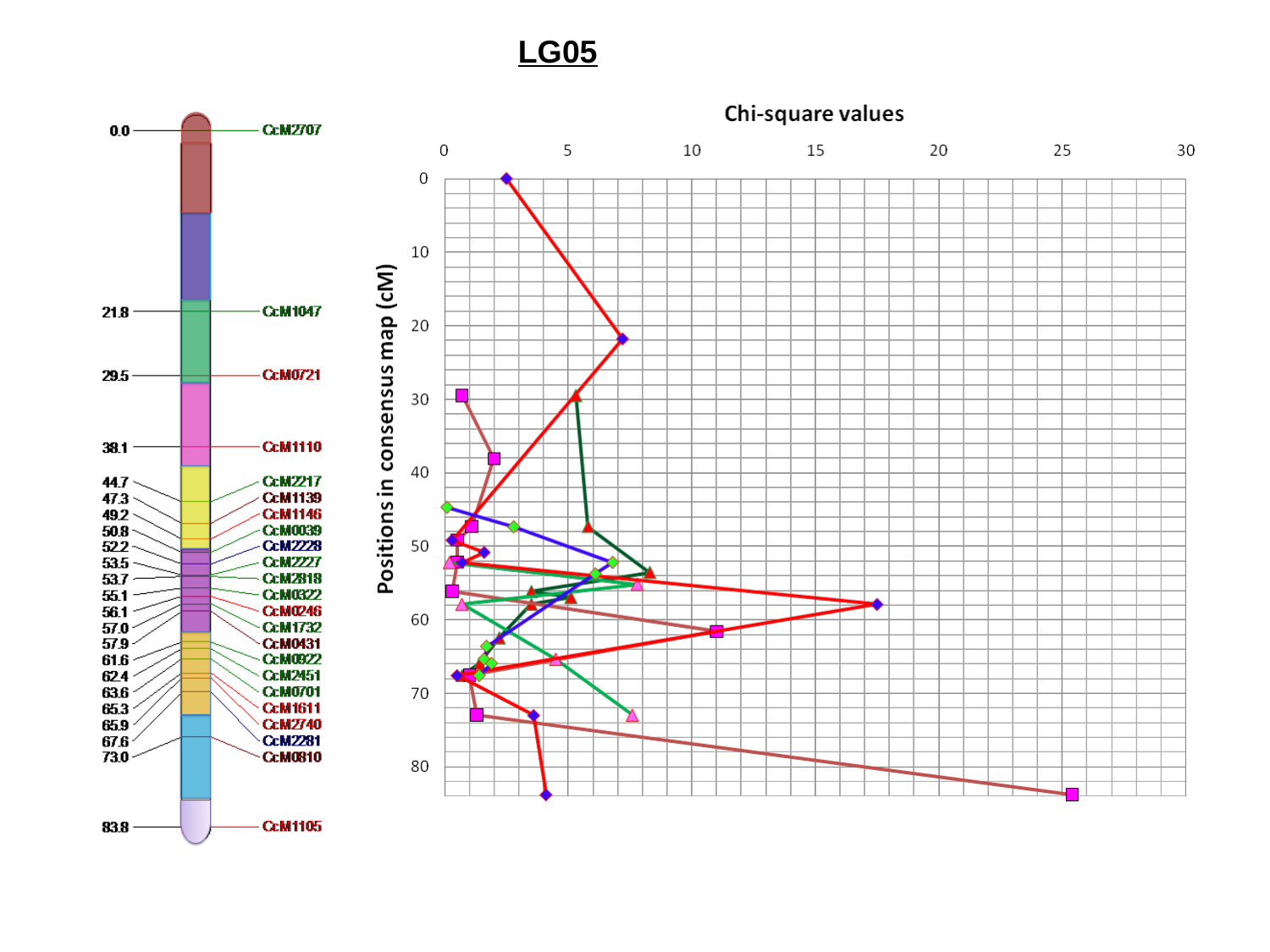

LG05

## Slide 6
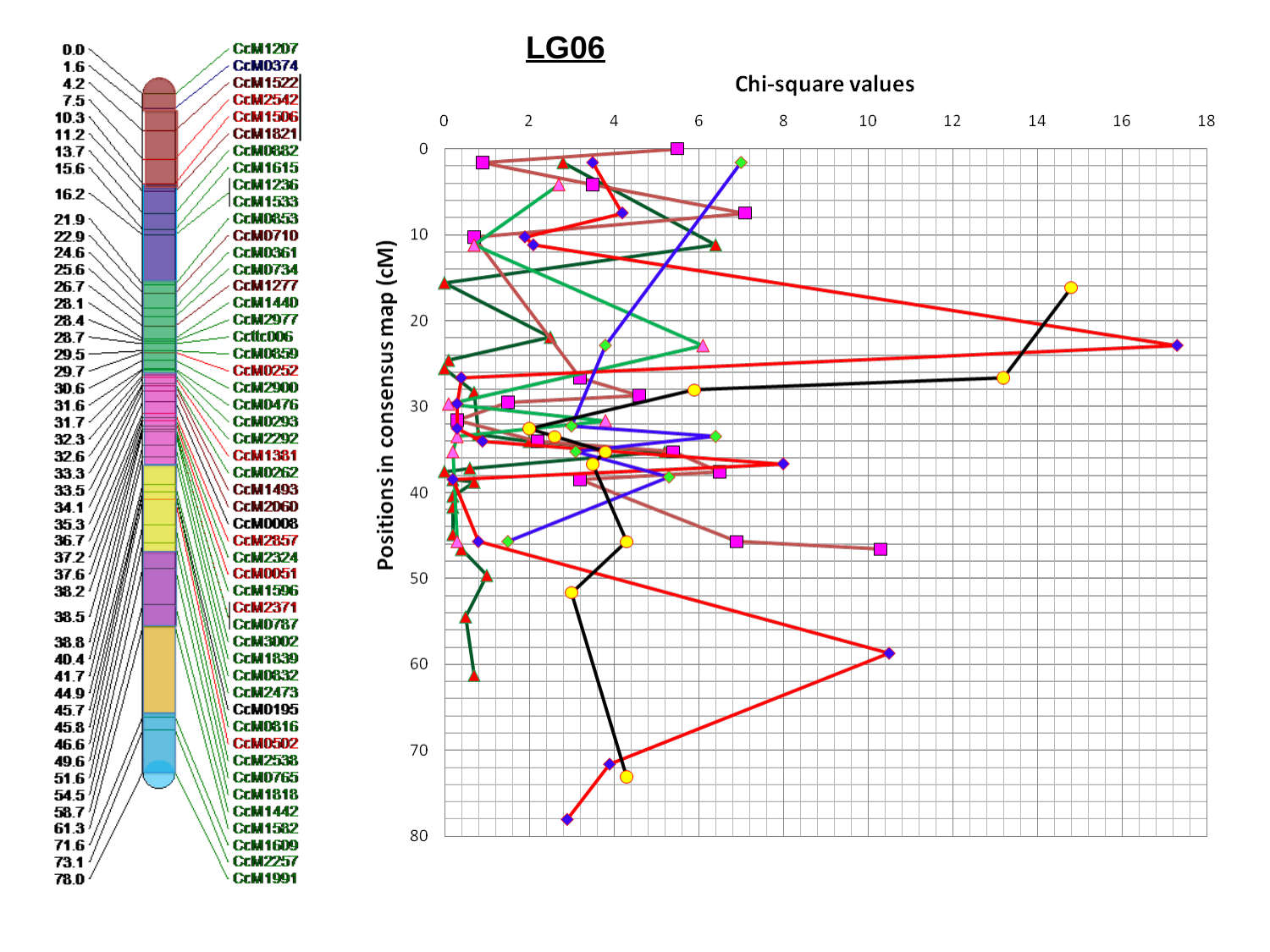

LG06

## Slide 7
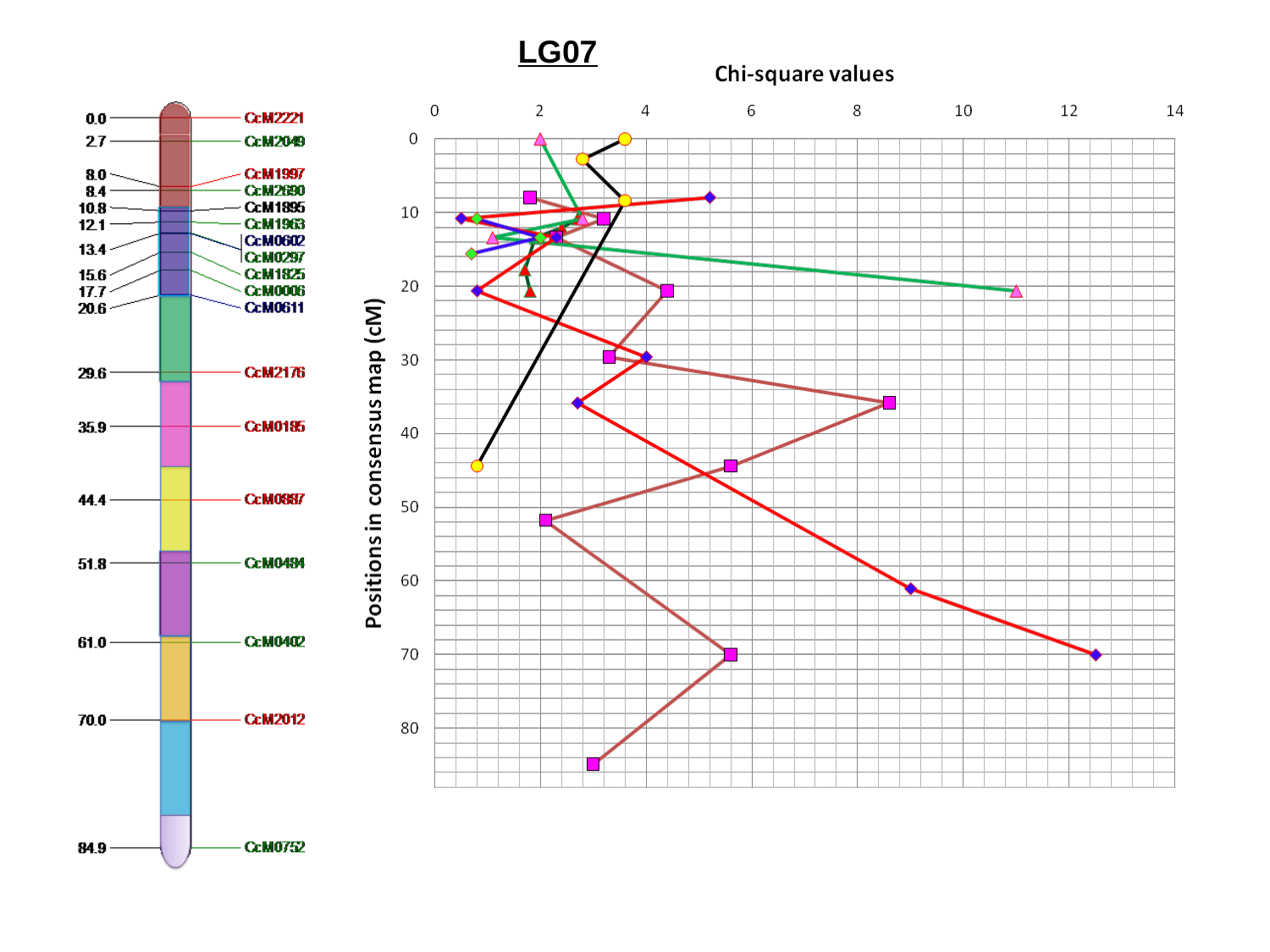

LG07

## Slide 8
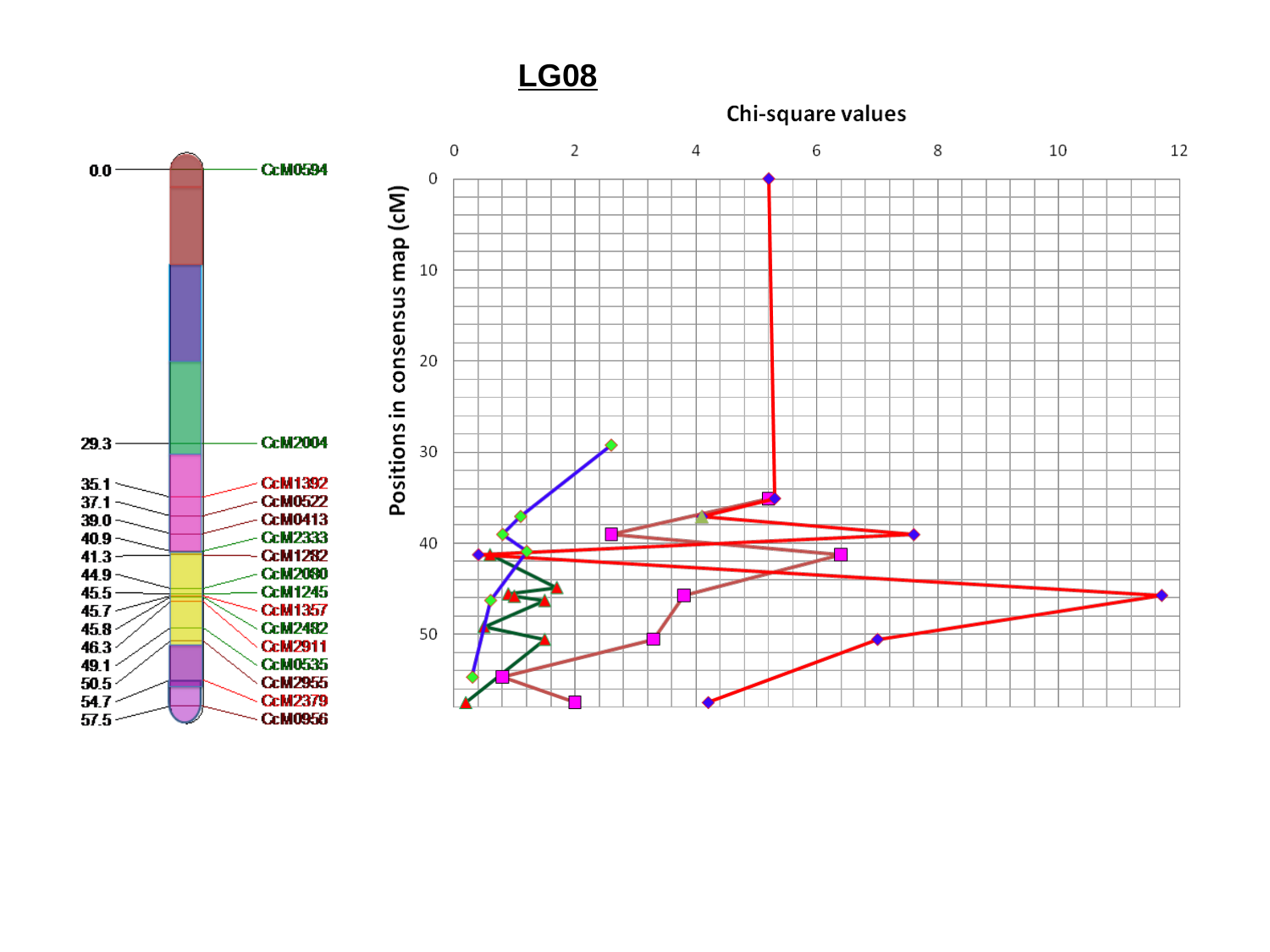

LG08

## Slide 9
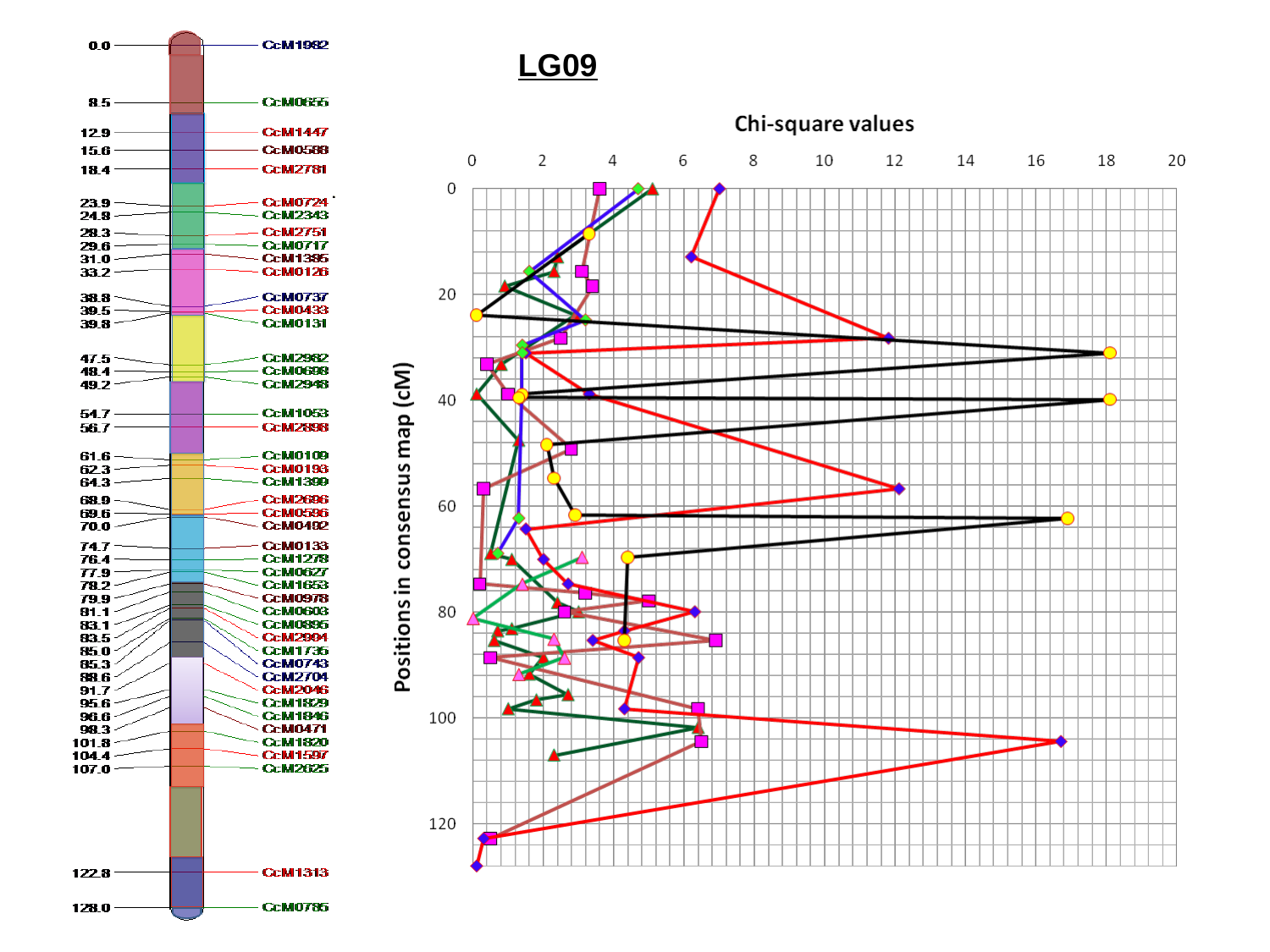

LG09

## Slide 10
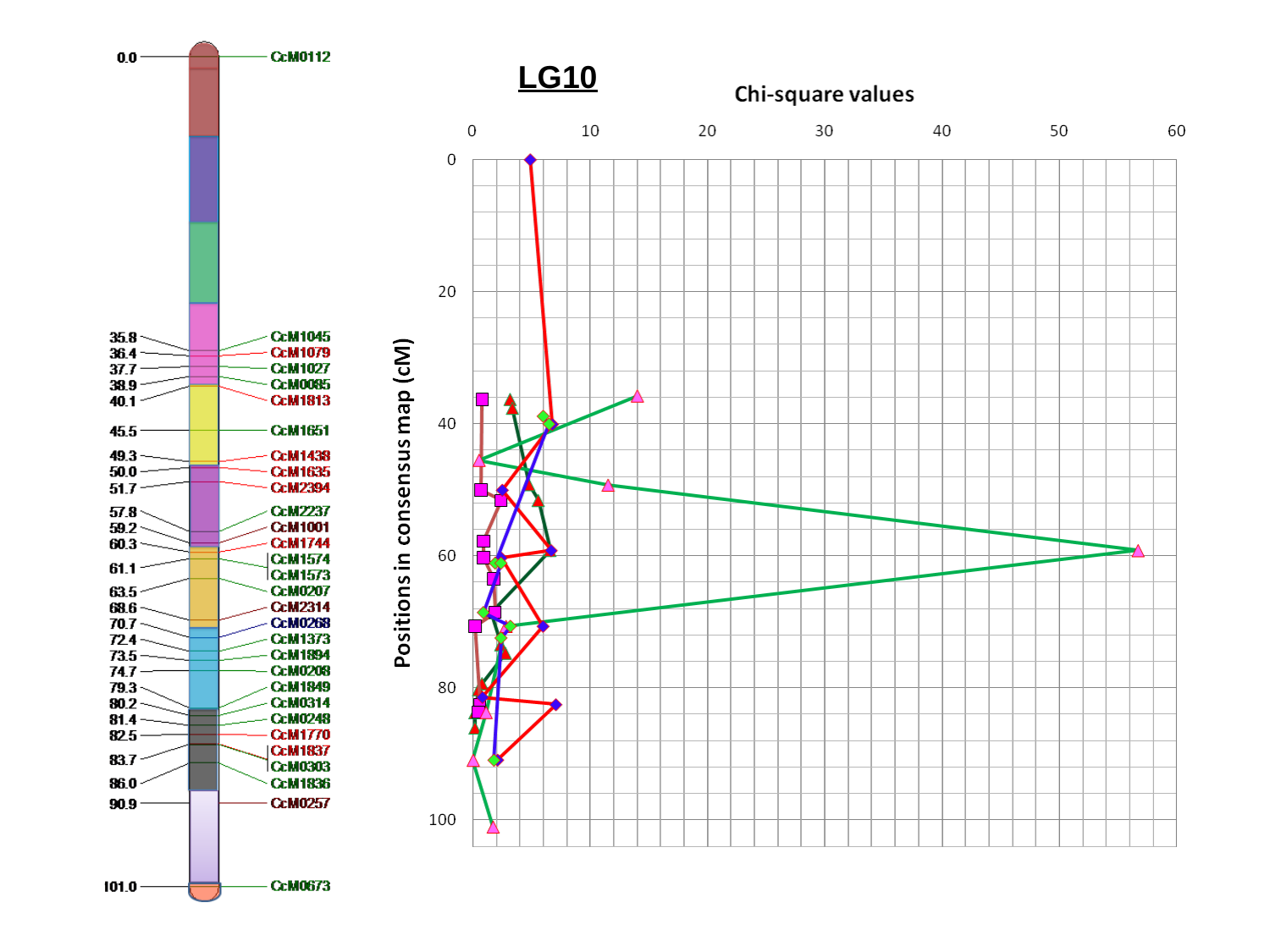

LG10

## Slide 11
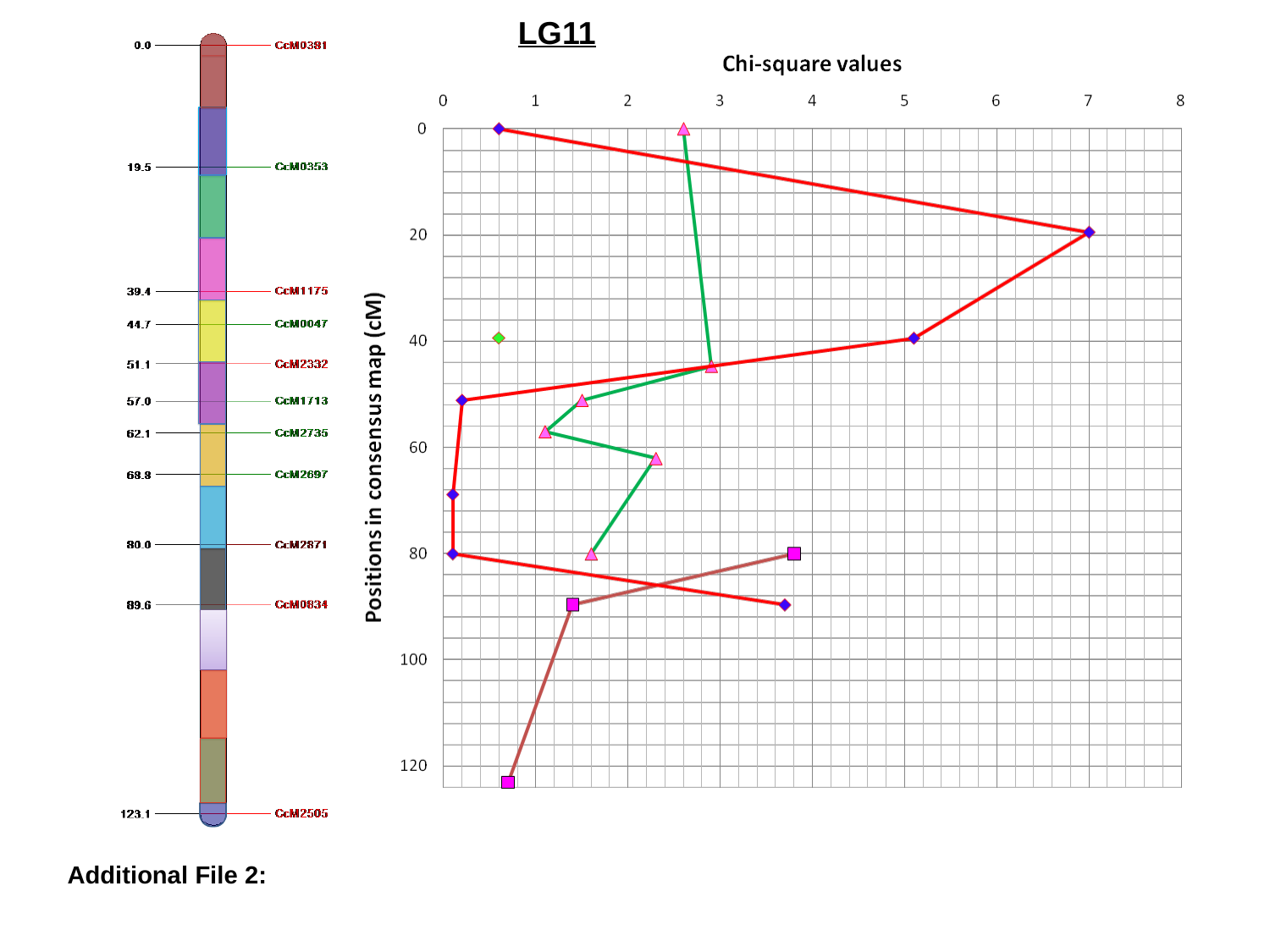

LG11
Additional File 2:
