# Supplementary material for: An intra-specific consensus genetic map of pigeonpea [Cajanus cajan (L.) Millspaugh] derived from six mapping populations
Source: Theor Appl Genet. 2012 Jul 8;125(6):1325–38. doi: 10.1007/s00122-012-1916-5 (PMC3442162; doi:10.1007/s00122-012-1916-5)
Supplement: Supplementary file 2 — Supplementary material 2 (PPT 628 kb) [file 122_2012_1916_MOESM2_ESM.ppt]

## Slide 1
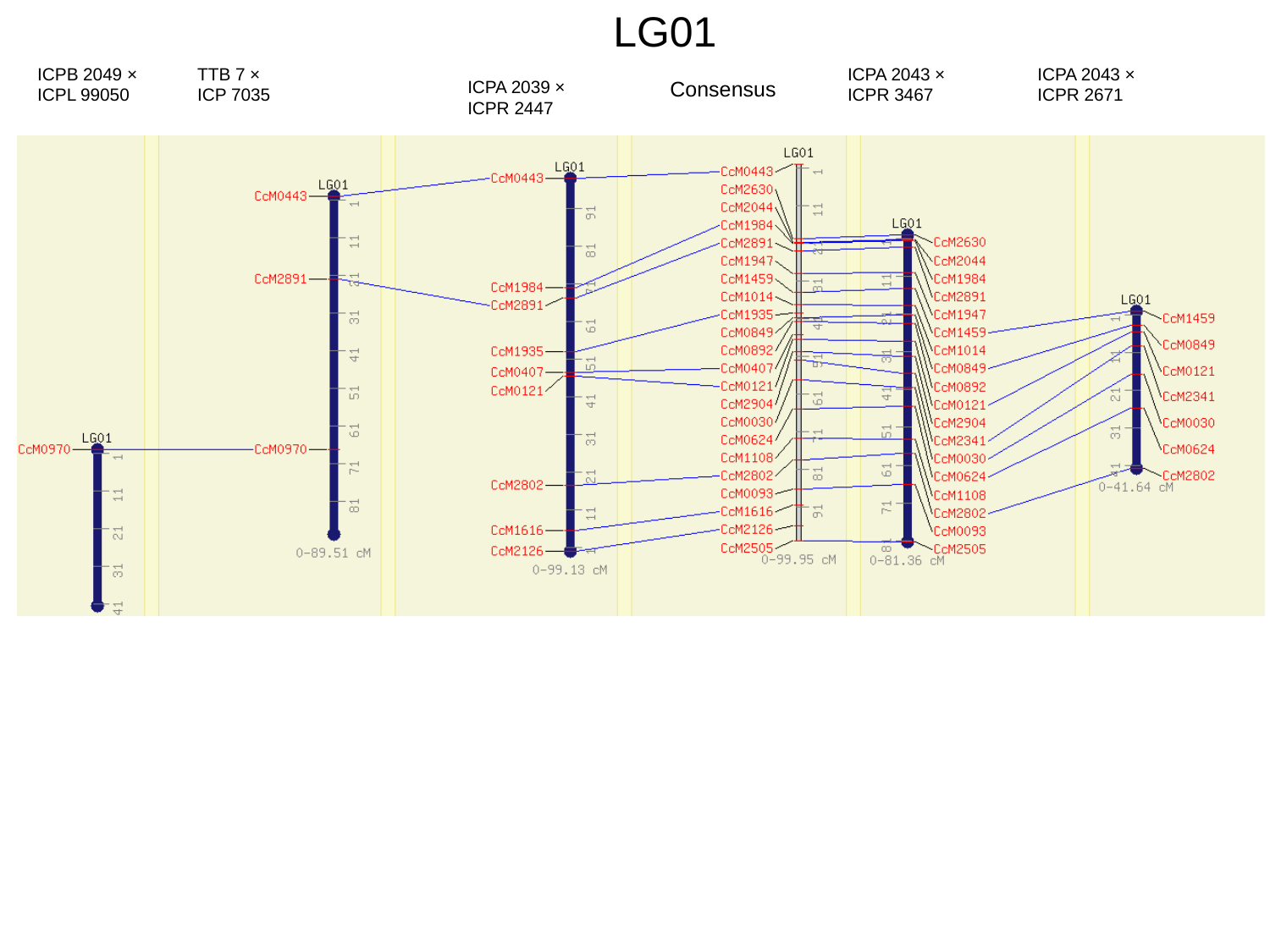

LG01
ICPB 2049 × ICPL 99050
TTB 7 × ICP 7035
ICPA 2043 × ICPR 3467
ICPA 2043 × ICPR 2671
Consensus
ICPA 2039 × ICPR 2447

## Slide 2
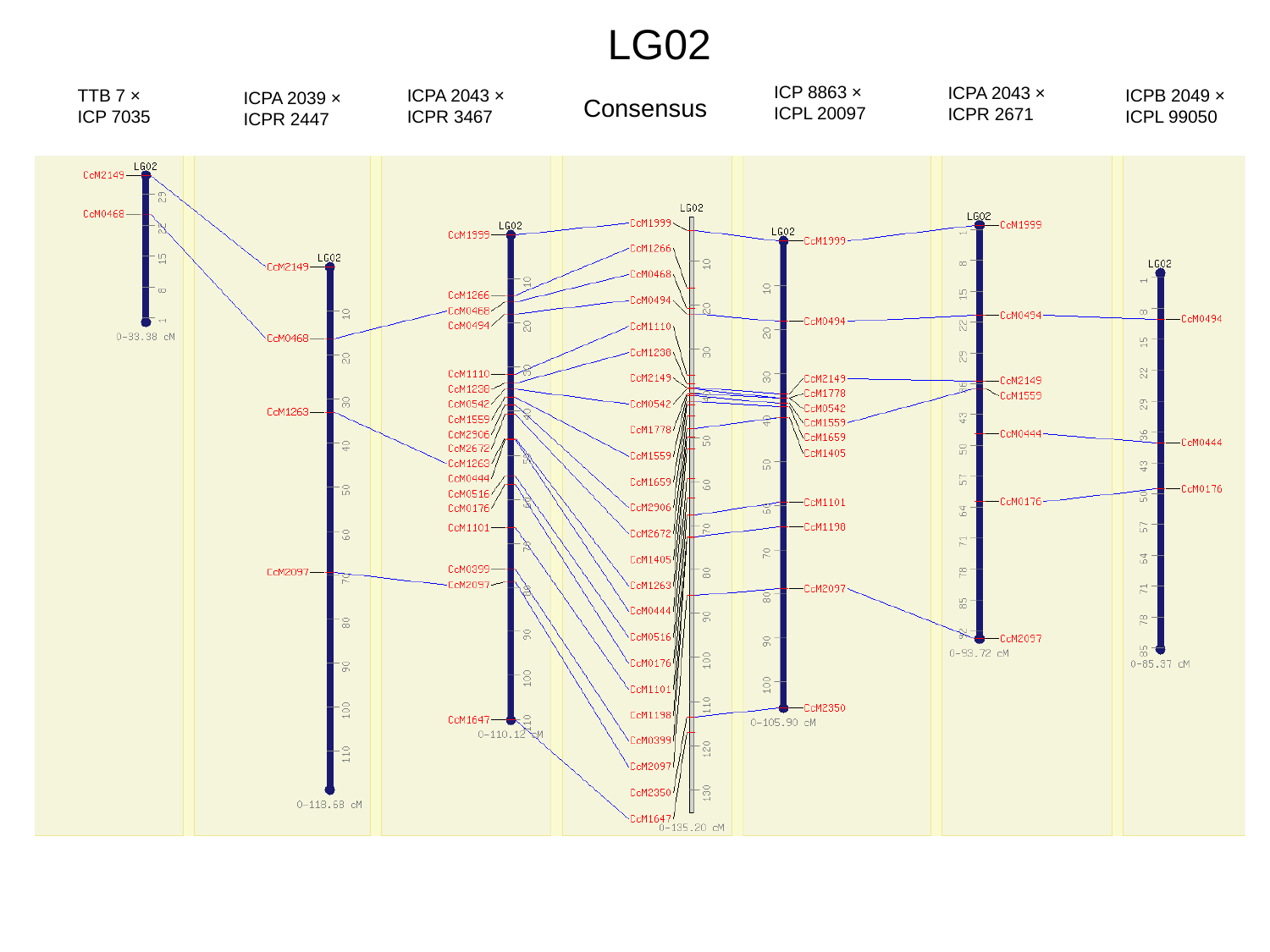

LG02
ICP 8863 × ICPL 20097
ICPA 2043 × ICPR 2671
TTB 7 × ICP 7035
ICPA 2043 × ICPR 3467
ICPB 2049 × ICPL 99050
ICPA 2039 × ICPR 2447
Consensus

## Slide 3
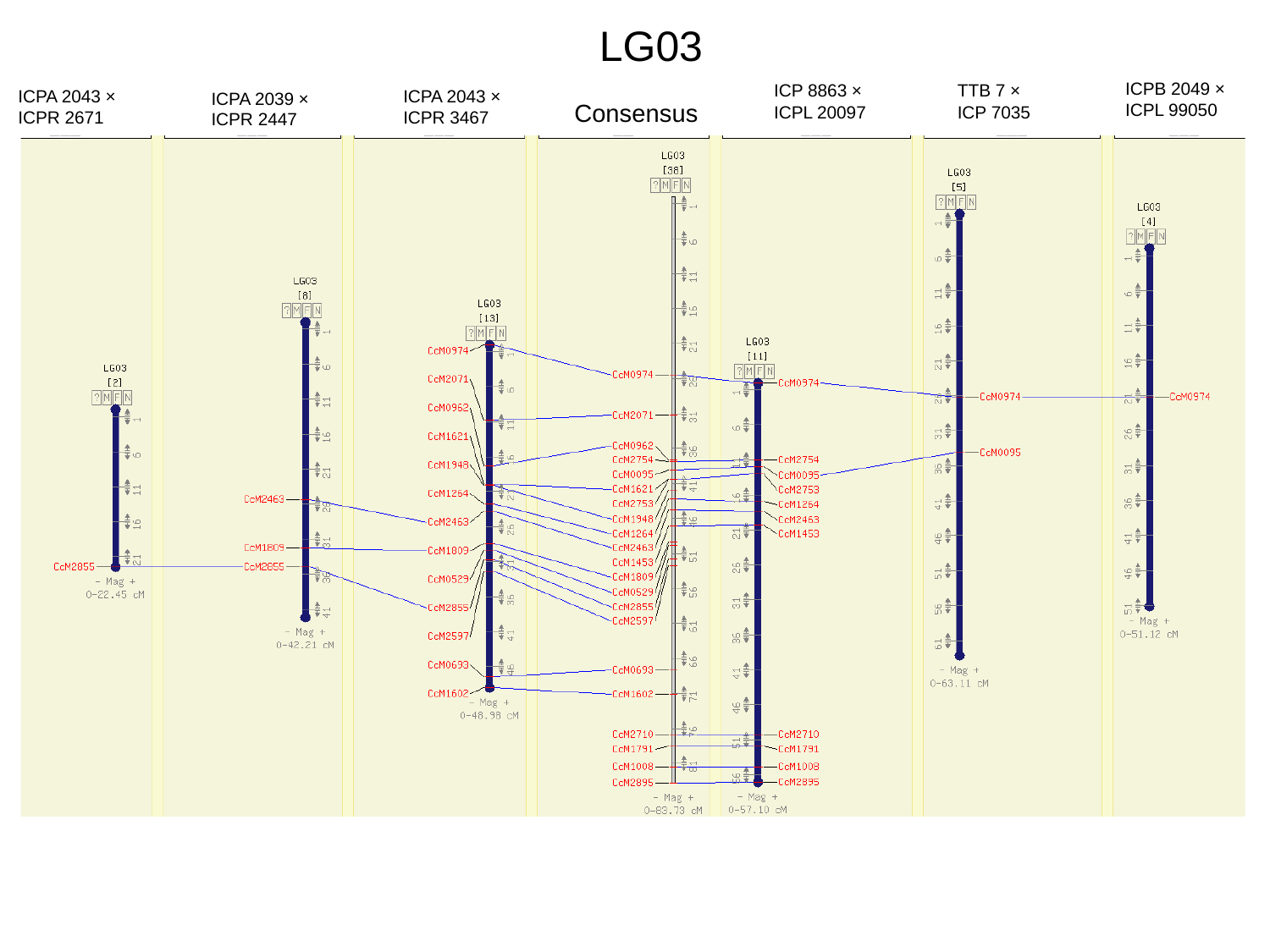

LG03
ICPB 2049 × ICPL 99050
ICP 8863 × ICPL 20097
TTB 7 × ICP 7035
ICPA 2043 × ICPR 2671
ICPA 2043 × ICPR 3467
ICPA 2039 × ICPR 2447
Consensus

## Slide 4
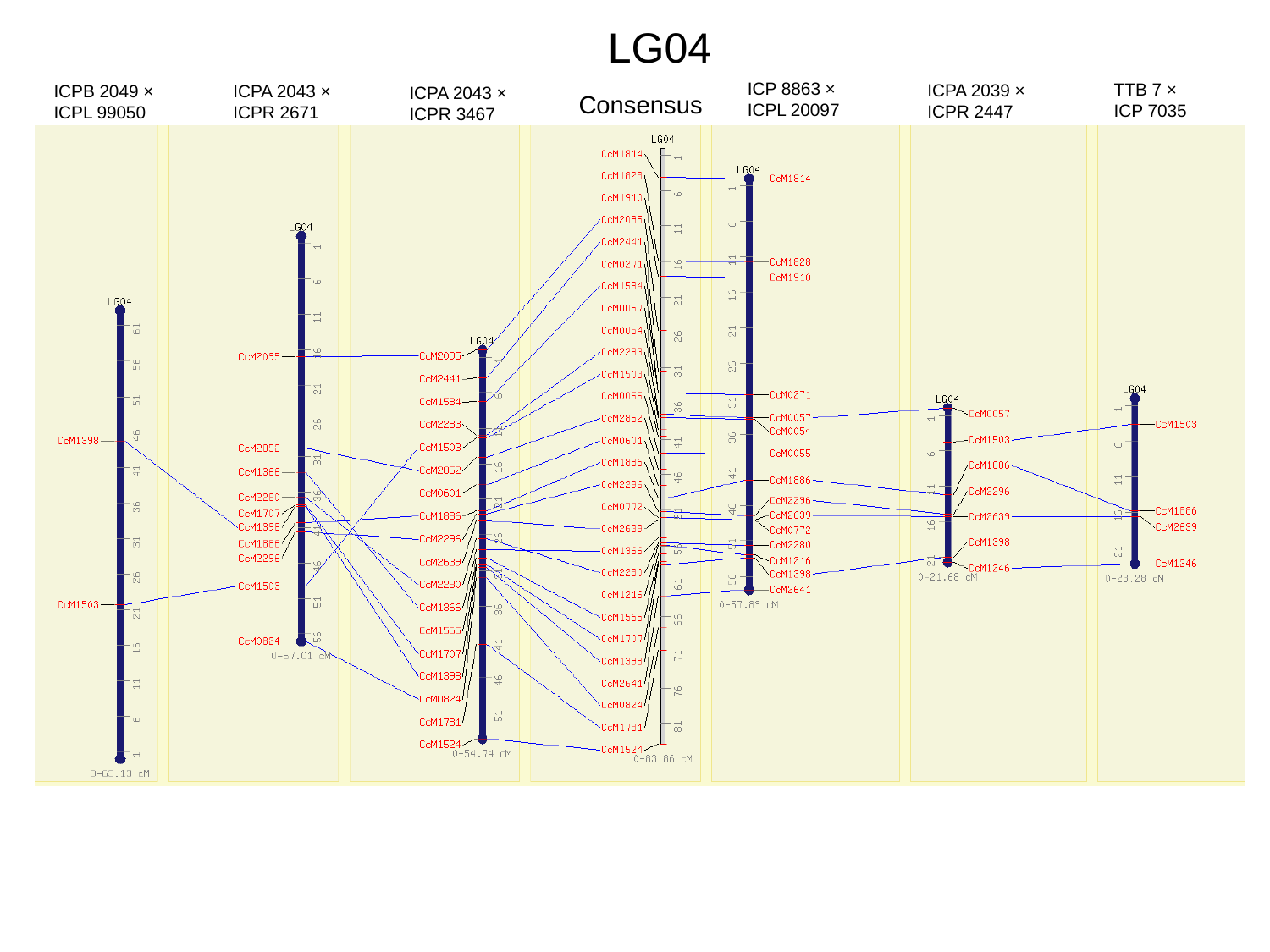

LG04
ICP 8863 × ICPL 20097
TTB 7 × ICP 7035
ICPA 2039 × ICPR 2447
ICPB 2049 × ICPL 99050
ICPA 2043 × ICPR 2671
ICPA 2043 × ICPR 3467
Consensus

## Slide 5
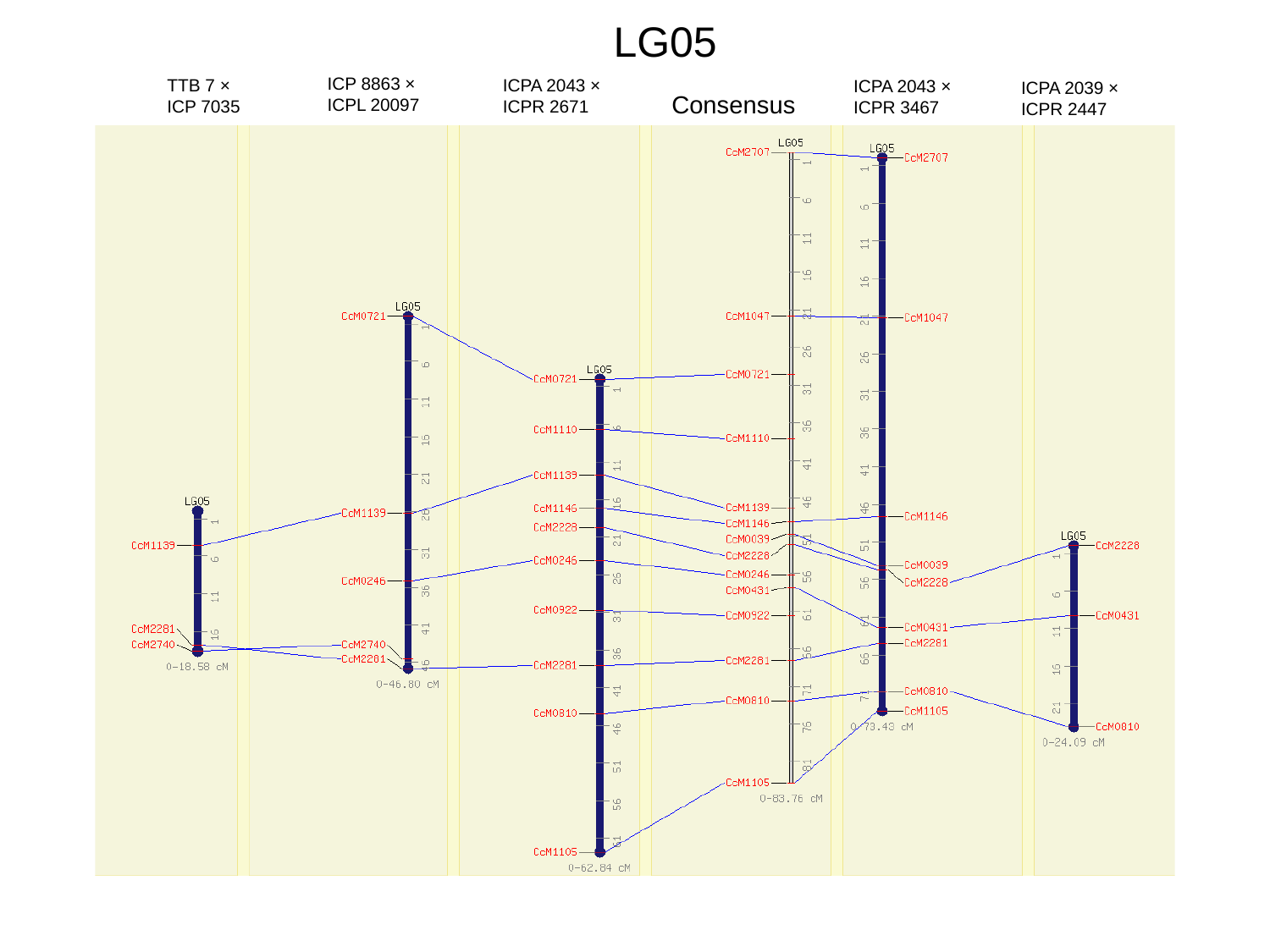

LG05
ICP 8863 × ICPL 20097
TTB 7 × ICP 7035
ICPA 2043 × ICPR 2671
ICPA 2043 × ICPR 3467
ICPA 2039 × ICPR 2447
Consensus

## Slide 6
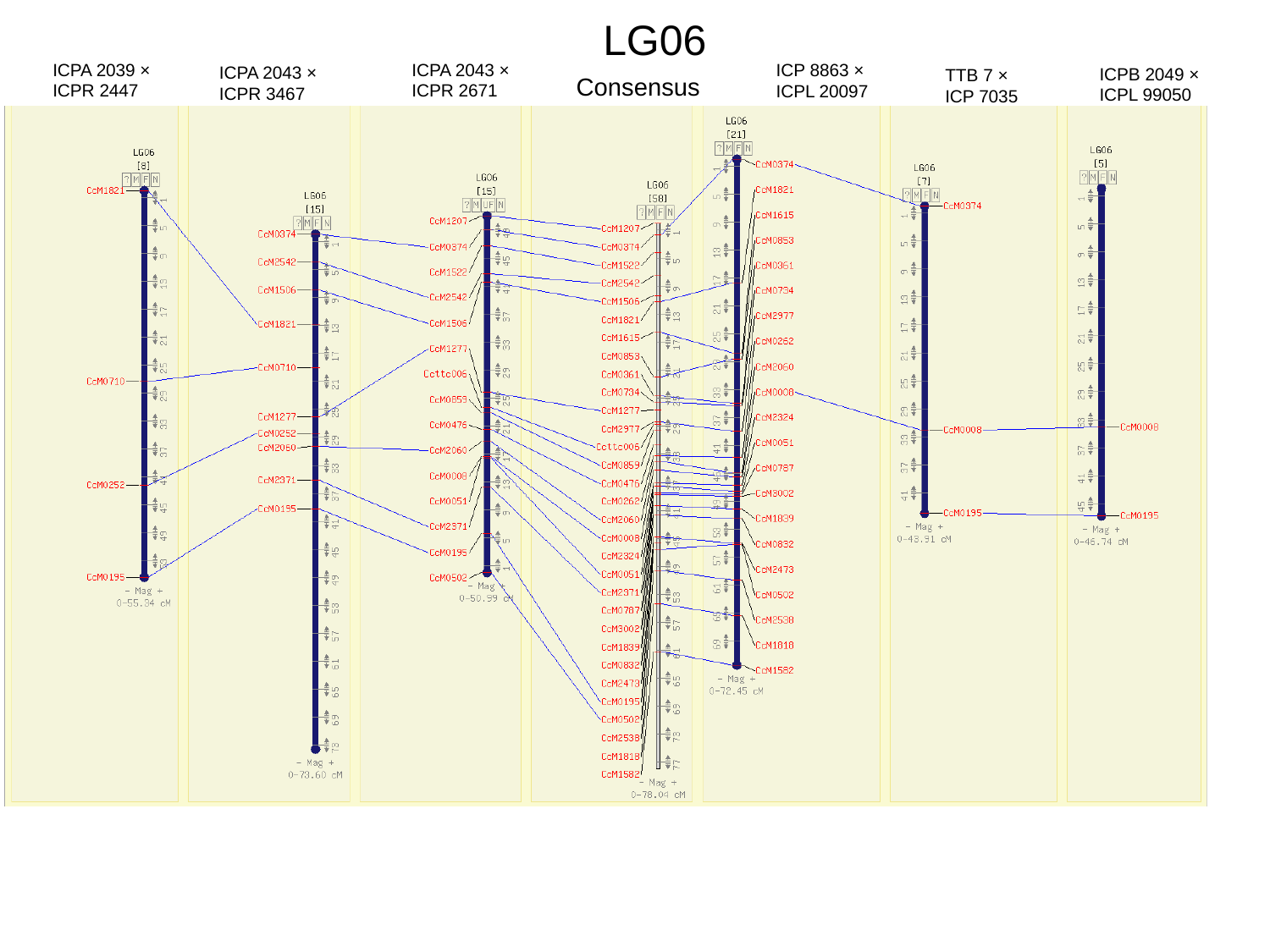

LG06
ICPA 2039 × ICPR 2447
ICPA 2043 × ICPR 2671
ICP 8863 × ICPL 20097
ICPA 2043 × ICPR 3467
ICPB 2049 × ICPL 99050
TTB 7 × ICP 7035
Consensus

## Slide 7
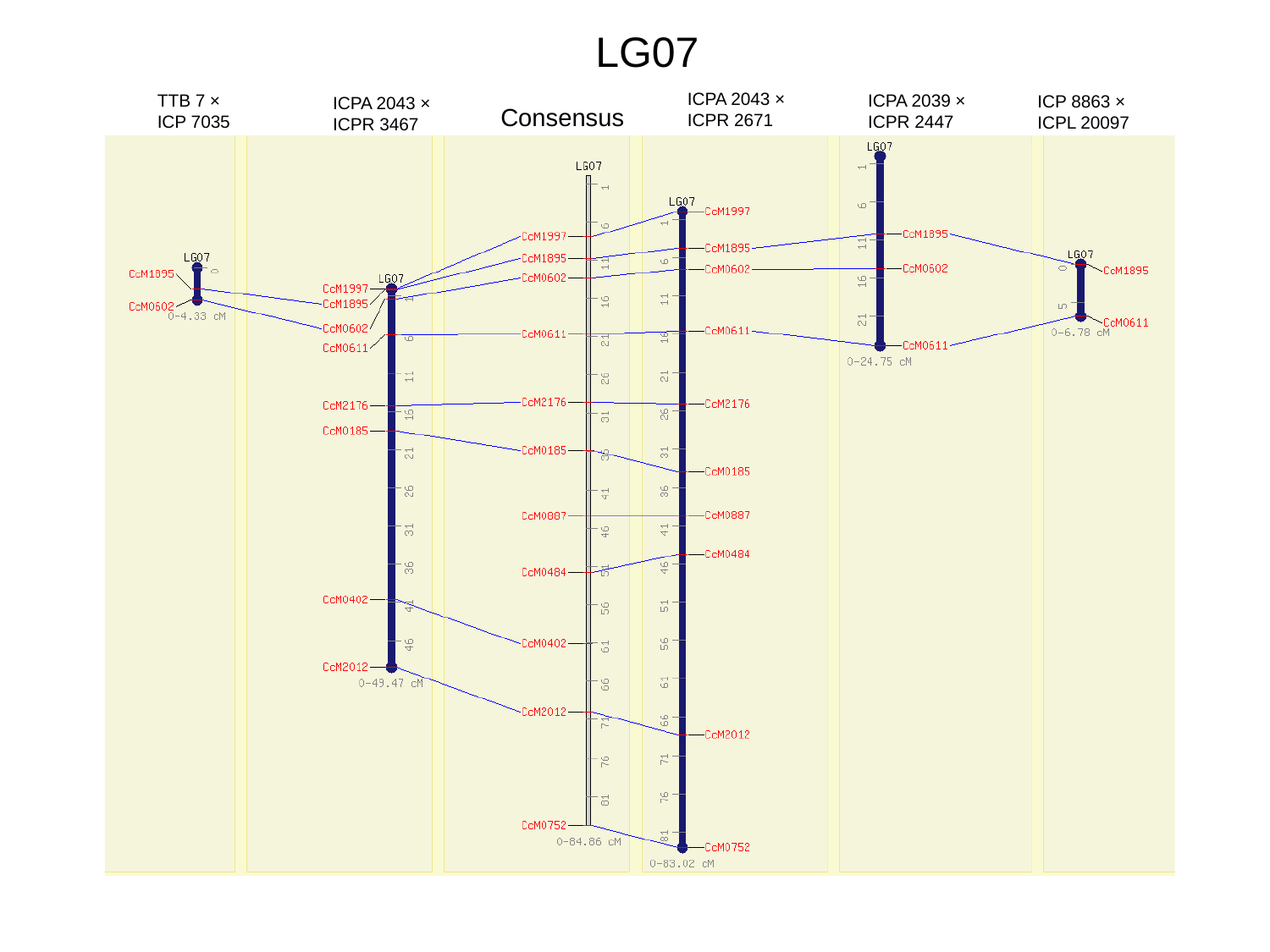

LG07
ICPA 2043 × ICPR 2671
ICPA 2039 × ICPR 2447
TTB 7 × ICP 7035
ICP 8863 × ICPL 20097
ICPA 2043 × ICPR 3467
Consensus

## Slide 8
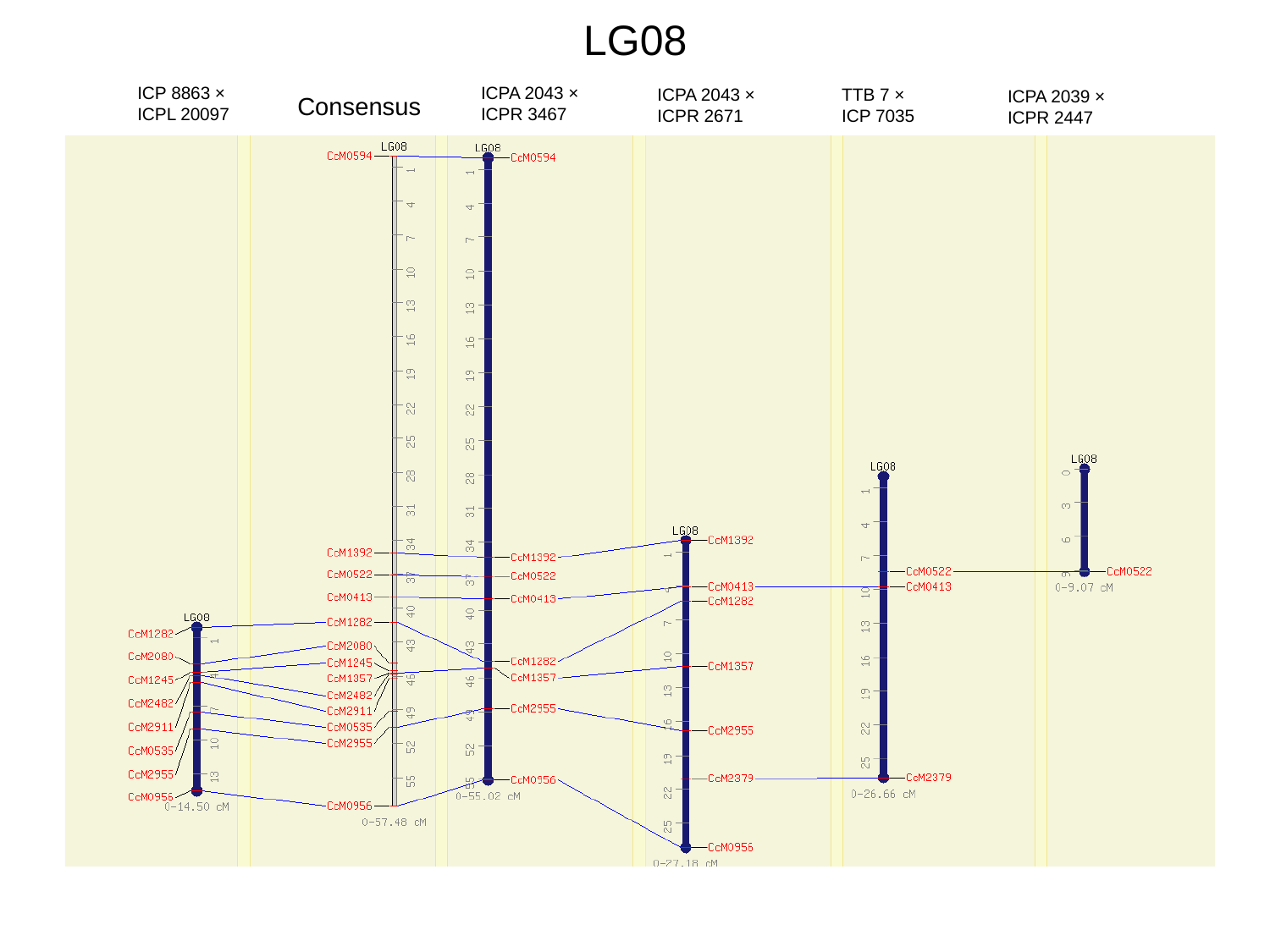

LG08
ICPA 2043 × ICPR 3467
ICP 8863 × ICPL 20097
TTB 7 × ICP 7035
ICPA 2043 × ICPR 2671
ICPA 2039 × ICPR 2447
Consensus

## Slide 9
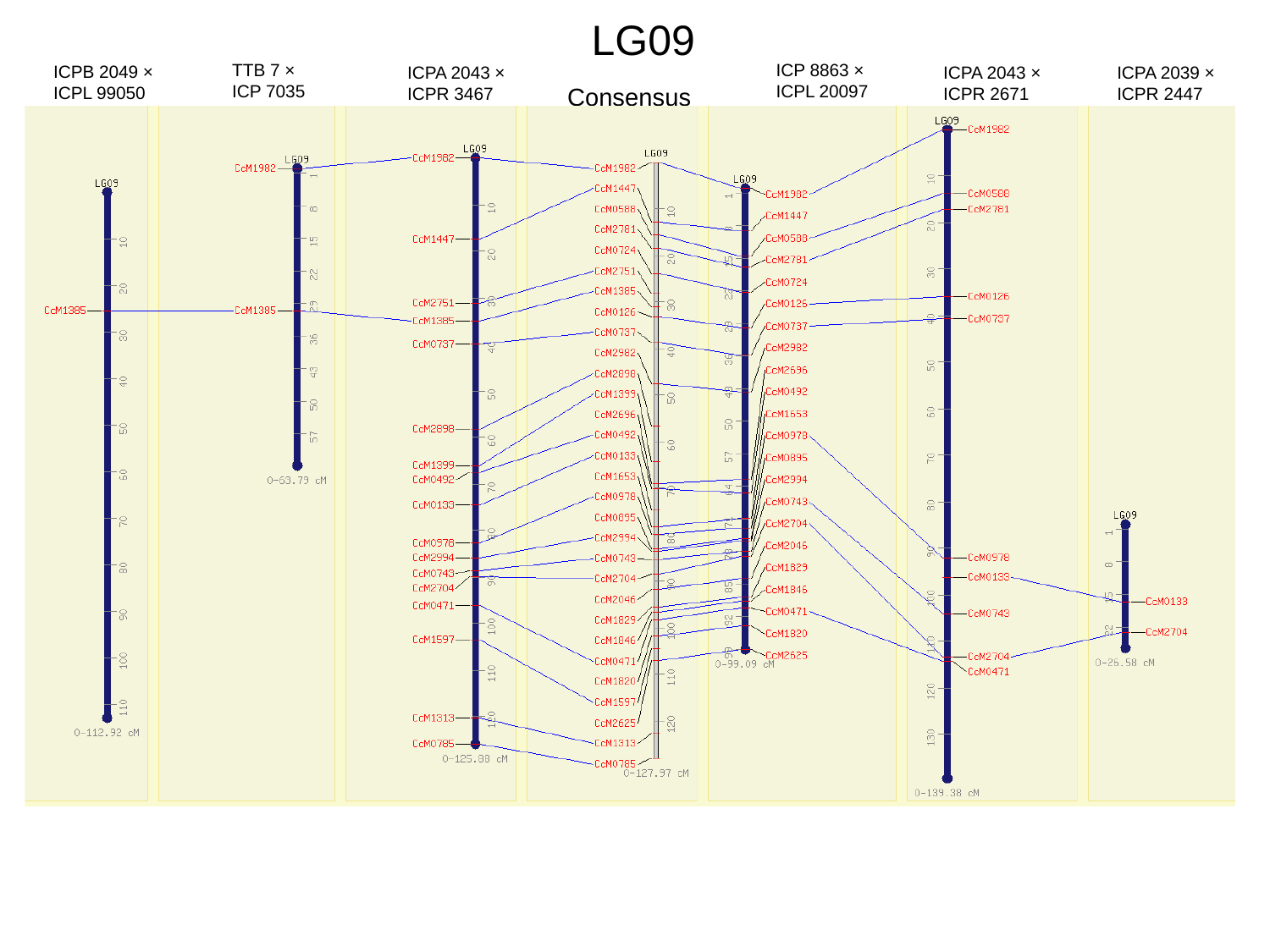

LG09
TTB 7 × ICP 7035
ICP 8863 × ICPL 20097
ICPB 2049 × ICPL 99050
ICPA 2043 × ICPR 3467
ICPA 2043 × ICPR 2671
ICPA 2039 × ICPR 2447
Consensus

## Slide 10
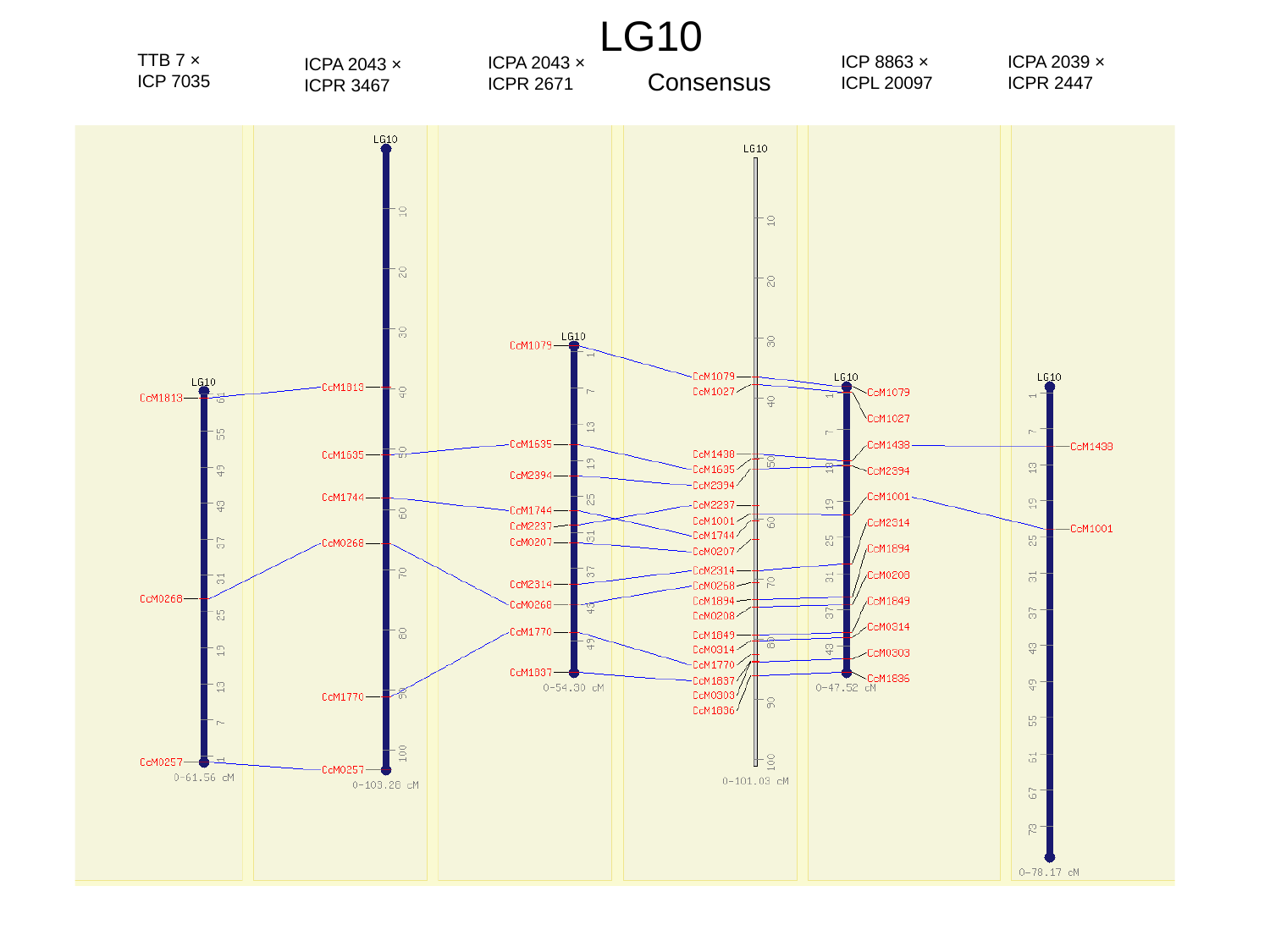

LG10
TTB 7 × ICP 7035
ICP 8863 × ICPL 20097
ICPA 2039 × ICPR 2447
ICPA 2043 × ICPR 2671
ICPA 2043 × ICPR 3467
Consensus

## Slide 11
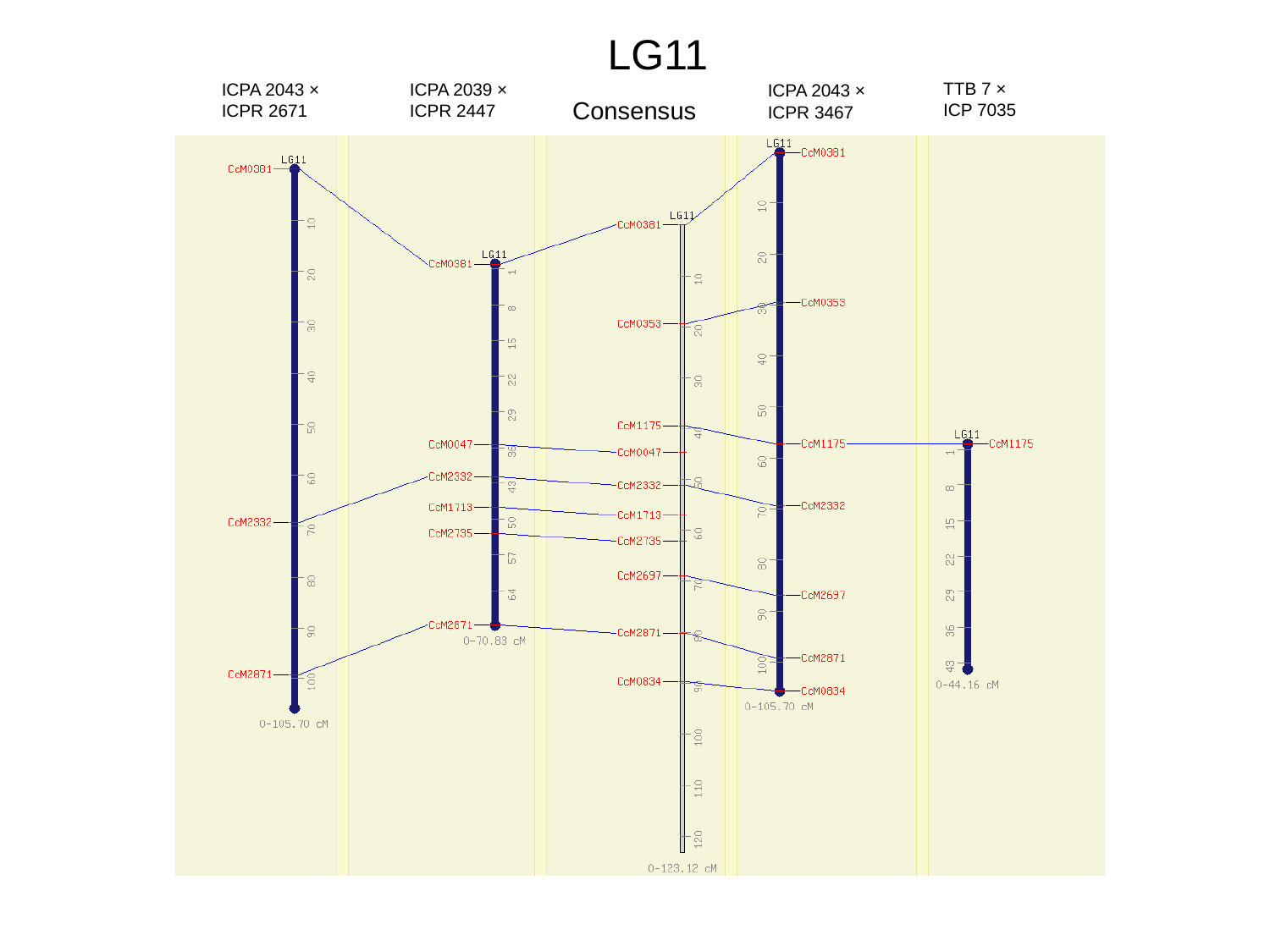

LG11
TTB 7 × ICP 7035
ICPA 2043 × ICPR 2671
ICPA 2039 × ICPR 2447
ICPA 2043 × ICPR 3467
Consensus
